# Supplementary material for: Coverage enhancement accelerates acidic CO2 electrolysis at ampere-level current with high energy and carbon efficiencies
Source: Nat Commun. 2024 Feb 24;15:1711. doi: 10.1038/s41467-024-45988-4 (PMC10894216; doi:10.1038/s41467-024-45988-4)
Supplement: Supplementary file 1 — Supplementary Information [file 41467_2024_45988_MOESM1_ESM.pdf]

Supplementary Information

Coverage enhancement accelerates acidic CO_2 electrolysis at ampere-level current with high energy and carbon efficiencies

Xiaohan Yu[1,†], Yuting Xu[2,†], Le Li[1,†], Mingzhe Zhang[1], Wenhao Qin[1], Fanglin Che[2,#,*], Miao Zhong[1,#,*]

[1]College of Engineering and Applied Sciences, National Laboratory of Solid State Microstructures, Collaborative Innovation Center of Advanced Microstructure, the Frontiers Science Center for Critical Earth Material Cycling, Nanjing University, Nanjing 210093, China.

[2]Department of Chemical Engineering, University of Massachusetts Lowell, Lowell, MA 01854, United States

[†]These authors contributed equally to this work.

[#]These authors jointly supervised this work: Fanglin Che, Miao Zhong.

[*]Correspondence and requests for materials should be addressed to Miao Zhong (miaozhong@nju.edu.cn), and Fanglin Che (Fanglin_Che@uml.edu).

Supplementary Methods details.

Synthesis. We fabricated a series of $Cu_{1-x}Sn_x$ (x = 0.08, 0.14, 0.28, 0.44, 0.71, 0.88), Cu, and Sn electrocatalysts by thermal evaporation (SKY-RH400). Cu and Sn particles were placed separately in two Molybdenum boats inside the deposition chamber, and melted slowly under the pressure of 10^{-5} Torr for thermal evaporation. We controlled the thermal evaporation rates of Cu and Sn to adjust the Cu/Sn ratios in the synthesized alloys. Evaporating rates of approximately x Å s^{-1} (x = 0.08, 0.14, 0.28, 0.44, 0.71, 0.88) for Sn and approximately ($1-x$) Å s^{-1} for Cu were used to produce alloyed $Cu_{1-x}Sn_x$ films on the polytetrafluoroethylene (PTFE) substrates. A quartz crystal monitor was used to observe the thicknesses of evaporated $Cu_{1-x}Sn_x$. Evaporation of pure Sn and pure Cu electrocatalysts was conducted using a similar fabrication procedure. The loading of all catalysts is 800 nm thick.

Characterization. Transmission electron microscopy (SEM) images were taken using a Hitachi SU8100 SEM at an accelerating voltage of 5 kV. High-resolution transmission electron microscopy (HRTEM) and transmission electron microscopy-energy dispersive X-ray spectroscopy (TEM-EDX), selected area electron diffraction (SAED), and bright-field and dark-field TEM analyses were performed in a TEM (Tecn F20) with an accelerating voltage of 200 kV. X-ray powder diffraction (XRD) was carried out with a Bruker D8 Advance at a scanning rate of 10° min^{-1} in the 2θ range from 20° to 80°. X-ray photoelectron spectroscopy (XPS) studies were performed using PHI5000 VersaProbe. The binding energy data were calibrated relative to the C 1s signal at 284.6 eV.

Electrochemical experiments. Experiments under alkaline and acidic conditions were performed in a flow cell using a three-electrode system. Ag/AgCl electrode was used as the reference electrode, commercial Ni foam (for use under alkaline conditions) and Pt (for use under acidic conditions) were used as the counter electrodes, $Cu_{1-x}Sn_x$ (x = 0.08, 0.14, 0.28, 0.44, 0.71, 0.88), Cu, and Sn on PTFE electrodes were used as the working electrodes (area: 0.5 cm^2), and the electrolytes were 1 M KOH (for alkaline

CO_2 electrolysis) and 0.05 M H_2SO_4 with 3 M KCl (for acidic CO_2 electrolysis). Anion exchange membrane (Fumasep FAB-PK-130, size: 2×2 cm^2, thickness: 130 μm) and proton exchange membrane (Nafion N117, size: 2×2 cm^2, thickness: 183 μm) were used as the ion exchange membranes. The proton exchange membrane was immersed in 0.5 M H_2SO_4 for about 2 hours before use. Experiments using solid-state electrolyte (SSE) were performed in a membrane electrode assembly (MEA) system with a 1 mm-thick SSE layer between the cathode and anode. IrO_x/Ti foam was used as the anode, and Cu_6Sn_5 was used as the cathode (area: 4 cm^2). Anion exchange membrane (Sustainion X37-50 Grade, size: 4×4 cm^2, thickness: 50 μm) and proton exchange membrane (Nafion N117, size: 4×4 cm^2, thickness: 183 μm) were used as the ion exchange membranes. The SSE was AmberChrom 50WX4 hydrogen form (J&K Scientific), and the anolyte was 0.5 M H_2SO_4. The electrochemical workstation was Autolab PGSTAT302N (Metrohm). The gas flow rate was controlled at 25 mL min^{-1} by an electronic flow meter, and the end flow rate was calibrated by a soap film flow meter during the test. The linear sweep voltammetry (LSV) studies were carried out with the potential range from 0 to −4 V_{RHE} (V vs. RHE). All of the electrode potentials vs. the Ag/AgCl electrode were converted to the potentials vs. reversible hydrogen electrode (RHE) using the following equation (1):

$$E_{RHE} = E_{Ag/AgCl} + 0.197 + 0.059 \times pH + iR \quad (1)$$

Gas-phase products were measured using gas chromatography (GC Agilent 990, Perkin Elmer Clarus 680). According to the peak area, the Faradaic efficiency of the gas products can be obtained, and the calculation formula is as the following equation (2):

$$FE = \frac{F \times z \times v \times n}{I \times t} \times 100\% \quad (2)$$

where F is the Faraday's constant, which is 96485 C mol^{-1}. z is the number of electrons required to reduce CO_2 to a CO or H_2 molecule, which is 2. v is the gas flow rate, here is 25 mL min^{-1}. n is the concentration of the gas products obtained by GC with 1 mL of sample gas, the unit is mol mL^{-1}. I is the current applied to the sample, the unit is A. t is the reaction time, and the unit is s.

Liquid product $HCOO^-$ was measured using ion chromatography (IC, SH-AC-11, Qingdao shenghan). The FE towards formate or FA at each current density was calculated by adding up both anodic and cathodic FEs. We first obtained a standard curve with a concentration gradient of sodium formate (HCOONa), $HCOO^-$ concentrations were set as 1 ppm, 2 ppm, 5 ppm, 10 ppm, 50 ppm, and 100 ppm (mg L^{-1}). According to this, the $HCOO^-$ concentration in the electrolyte after the reaction can be obtained. The formula of faradaic efficiency calculation for $HCOO^-$ is as the following equation (3):

$$FE = \frac{F \times z \times V \times n}{45 \times I \times t} \times 100\% \tag{3}$$

where F is the Faraday's constant, which is 96485 C mol^{-1}. n is the concentration of $HCOO^-$ measured by the instrument based on the standard curve, and the unit is mg L^{-1}. z is the number of electrons required for the reduction of CO_2 into $HCOO^-$, here is 2. V is the volume of the catholyte, here is 0.03 L. t is the reaction time, and the unit is s.

The cathode energy efficiency (CEE) in a flow cell was calculated using the following equation (4):

$$CEE = \frac{(1.23 - E_{HCOOH}) \times FE_{HCOOH}}{1.23 - E_{cathode}} \tag{4}$$

Where E_{HCOOH} is −0.199 V_{RHE} to form HCOOH in aqueous electrolytes. FE_{HCOOH} is the Faradaic efficiency of HCOOH. $E_{cathode}$ is the applied cathode potential (V vs. RHE).

The full-cell energy efficiency (EE) in a SSE-based MEA was calculated using the following equation (5):

$$EE = \frac{(1.23 - E_{HCOOH}) \times FE_{HCOOH}}{E_{cell}} \tag{5}$$

Where E_{HCOOH} is −0.199 V to form HCOOH in aqueous electrolytes. FE_{HCOOH} is the Faradaic efficiency of HCOOH. E_{cell} is the applied full-cell potential (V).

The SPCE was calculated using the following equation (6):

$$SPCE = \frac{(I_{HCOOH} \times 60\, s)/(N \times F)}{(v \times 1\ \mathrm{min})/(24.05\, L\, mol^{-1})} = \frac{n_{HCOOH}}{n_{CO_2}} \tag{6}$$

Where I_{HCOOH} represents the partial current of HCOOH in amperes, N stands for the electron transfer which is 2 for HCOOH, F is Faraday's constant, which is 96485 C mol^{-1}, v is the flow rate of CO_2 in L min^{-1}.

All data, including Faradaic efficiencies, were collected based on 1-hour electrolysis. Stability tests were conducted over 300 hours. The overpotential is determined by subtracting the operating potential at specific current densities for formic acid generation from the theoretic potential for formic acid production, which is −0.199 V_{RHE}.

In situ electrochemical attenuated total reflection Fourier-transform infrared spectroscopy (ATR-FTIR) experiments were conducted on a Thermo Scientific Nicolet 6700 FTIR spectrometer with ZnSe as the prismatic window at room temperature. A three-electrode electrochemical single-cell was used for the tests (Supplementary Fig. 57). The thermally-evaporated Cu_6Sn_5 catalyst on a carbon gas diffusion layer (Freudenberg H15C13) was used as the working electrode, a Pt wire was used as the counter electrode, and a saturated Ag/AgCl electrode was used as the reference electrode. 3 M KCl and 0.05 M H_2SO_4 (pH = 1) saturated with CO_2 was used as the electrolyte. Open circuit potential (OCP) was conducted as a comparison, and the data was collected using chronoamperometric tests from −0.24 to −1.64 V_{RHE}. The peak area of *OCHO in FTIR was calculated by integrating the corresponding curve areas in the same interval (1250–1500 cm^{-1}) obtained at different potentials.

Computational Setup. DFT calculations were conducted using the Vienna *Ab-initio* Simulation Package (VASP).[1] These calculations were used to compare the energy diagrams of two possible CO_2 reduction reaction (CO_2R) pathways over different surfaces. According to XRD results from experimental characterization, we have investigated Cu (111), $Cu_{0.86}Sn_{0.14}$ (111), Cu_6Sn_5 (−113), and Sn (100) surfaces for calculations (Supplementary Fig. 4), where these facets are also the most thermodynamic favorable ones for Cu, Sn, and $Cu_{1-x}Sn_x$ alloy systems. For the $Cu_{0.86}Sn_{0.14}$ alloy system, we have displaced one Cu atom to Sn atom for each layer of Cu (111), that is to say, the Sn/Cu ratio in our theoretical model is nearly 12.5%. As the

percentage of Sn in the alloy system is close to the experimental case, this model can represent the $Cu_{0.86}Sn_{0.14}$ alloy system.

For all calculations we used revised Perdew-Burke-Ernzerhof (RPEB) exchange-correlation functional.[2] The lattice constants of pure Cu is calculated as 3.658 Å and lattice constant of pure Sn is a=b=4.48 Å and c=2.82 Å using RPEB, which are similar with the literature results.[3,4] For the surfaces mentioned above, the surface formation energy can be calculated with following equations[5]:

$$E_{sur}=\frac{E_{total}}{2A}=\frac{E_{slab}-N{\cdot}E_{bulk}}{2A} \tag{7}$$

$$E_{sur}=\frac{E_{total}}{2A}=\frac{E_{slab}-N_{Cu}{\cdot}E_{Cu\text{-}bulk}-N_{Sn}{\cdot}E_{Sn\text{-}bulk}}{2A} \tag{8}$$

where pure Cu, Sn, and Cu_6Sn_5 (−113) surfaces can be calculated with Equation (7). E_{slab} and E_{bulk} denote the energy of the energy of the total surfaces and bulk energy of Cu in the FCC structure, Sn in the tetragonal structure and Cu_6Sn_5 alloy in monoclinic structure,[6,7,8] respectively. A is the surface area and N is the number of atoms in the surfaces. The surface formation energy of Cu-Sn alloy surfaces can be calculated with Equation (8). E_{Cubulk} and E_{Snbulk} denote the bulk energy of Cu and Sn atoms, respectively. The $Cu_{0.86}Sn_{0.14}$ (111) surface corresponds to the Cu (111) surface with Sn atoms introduced as dopants. The values for E_{Cubulk} and E_{Snbulk} in this context are derived from calculations based on pure Cu and Sn bulk materials. Our results on the surface formation energy of Cu (111) agree well with previous investigations, in which the surface formation energy of the Cu (111) surface in our study is determined to be 0.98 J/m^2 and the corresponding value in the open literature derived from DFT calculations is reported to be 1.17 J/m^2.[9]

As shown in Supplementary Fig. 39, compared to the surface formation energy of Cu (111), it is evident that the Cu_6Sn_5 (−113) surface is more stable with a notably low surface formation energy. While the Sn (100) surface is less stable with a higher surface formation energy than that of Cu (111). These results agree well with the experiments regarding the great stability of the Cu_6Sn_5 catalyst and the poor stability of the Sn catalyst during prolonged acidic CO_2R.

To construct the energy diagram, we calculated the adsorption of CO_2R-to-CO or -HCOOH related intermediates at various adsorption sites over the above-mentioned surfaces and identified the most favorable configuration for each species.

Adsorption calculations. In this work, we investigated two possible reaction pathways from CO_2 to formic acid (FA) and CO, as shown in Supplementary Fig. 3. For pathway A, the first proton transfer to CO_2 forms *OCHO as the key intermediate where two O atoms bond with the surfaces. Then FA can be formed after the second proton transfer to *OCHO. As for pathway B, *COOH would be the intermediate for CO production. We have examined the species in these two pathways over four different surfaces (Cu (111), $Cu_{0.86}Sn_{0.14}$ (111), Cu_6Sn_5 (−113), and Sn (100)). After identifying the most favorable adsorption configurations for each involved species over each surface, we then built the energy diagram of CO_2R-to-CO and -HCOOH for each surface. The adsorption energy (E_{ad}) can be calculated according to the following equation:

$$E_{ad} = E_{total} - E_{slab} - E_{adsorbate} \quad (9)$$

where E_{total} denotes the total energy of the species over the surface, E_{slab} denotes the energy of the surface, and $E_{adsorbate}$ denotes the energy of adsorbate in the gas phase. The possible adsorption sites for each adsorbate on Cu (111) surface include top, bridge, fcc, and hcp, as shown in Supplementary Fig. 6.

For the $Cu_{0.86}Sn_{0.14}$ (111) surface, the possible adsorption sites include fcc, hcp, top and bridge sites of Cu atoms, top site of Sn atoms, and interface sites among Cu and Sn atoms for different adsorbates, as shown in Supplementary Fig. 7.

Over the Cu_6Sn_5 (−113) surface, the possible adsorption sites for each adsorbate include the bridge and top sites of Cu atoms, the bridge and top sites of Sn atoms, and the interface sites among the surfaces, as shown in Supplementary Fig. 8.

For the Sn (100) surface, the possible adsorption sites include top and bridge sites of upper and lower Sn atoms for each adsorbate, as shown in Supplementary Fig. 9.

For each surface, we have examined all of the adsorbates that participated in CO_2R-to-C_1 pathways with possible adsorption configurations and corresponding adsorption energy. The possible adsorption configurations of CO_2, *CO, *COOH,

*OCHO, HCOOH, and H_2O over Cu (111) are given in Supplementary Figs. 10–15.

The possible adsorption configurations of CO_2, *CO, *COOH, *OCHO, HCOOH, and H_2O over $Cu_{0.86}Sn_{0.14}$ (111) are given in Supplementary Figs. 16–21.

The possible adsorption configurations of CO_2, *CO, *COOH, *OCHO, HCOOH, and H_2O over the Cu_6Sn_5(−113) surface are given in Supplementary Figs. 22–27.

The possible adsorption configurations of CO_2, CO, *COOH, *OCHO, HCOOH, and H_2O over the Sn (100) surface are given in Supplementary Figs. 28–33.

Gibbs free energy calculations. In our work, the Gibbs free energy of each step (ΔG) can be obtained with the following equation using DFT calculations:[10]

$$\Delta G = \Delta E_{ad} + \Delta ZPE - T\Delta S + \Delta G_U + \Delta G_{pH} \quad (10)$$

where ΔE_{ad} gives the energy difference between the initial state and final state obtained from DFT calculations. ΔZPE is the zero-point energy difference, which can be calculated with equation (11):[11]

$$ZPE = \frac{1}{2}\sum_i hv_i \quad (11)$$

where v_i gives the vibrational frequencies for species adsorbed over the surfaces, which can be obtained within DFT calculations. Entropy (S) of the gas phase molecules, such as CO_2 can be obtained from NIST-JANAF thermodynamical tables.[12]

Since the partition function for the translational and rotational degrees of freedom for surface species becomes frustrated-translational-vibrational, frustrated-rotational-vibrational degrees of freedom, the entropy (S_{vib}) of surface species was calculated with the contribution with vibrational entropy only. The S_{vib} of surface species was calculated using equation (12): [13, 14]

$$S_{vib} = R\sum_i [\frac{hv_i}{k_B T}\frac{e^{-\frac{hv_i}{k_B T}}}{1 - e^{hv_i}} - ln(1 - e^{-\frac{hv_i}{k_B T}})] \quad (12)$$

where k_B denotes the Boltzmann constant. ΔG_U and ΔG_{pH} give the correction from applied potential and pH to the reaction-free energy, respectively. The energetics under different applied potentials can be corrected as:[15]

$$\Delta G_U = -eU \quad (13)$$

where U refers to the electrode potential. The pH correction can be given by:[16]

$$\Delta G_{pH} = -k_B T \, ln(|^*H|) = -0.0592pH \quad (14)$$

In our theoretical work about CO_2R, ΔG can be calculated at standard condition (P = 1 bar, T = 298.15 K) where the applied potential of −2.1 V_{RHE} and pH of 1 are consistent with the experimental conditions. To validate our calculation work, we have compared the calculation results with that in the open literature about two possible CO_2R pathways over Cu (111) surface and agree well with the reported literature.[17]

Hydrogen evolution reaction. We also examined the hydrogen evolution reaction (HER), which is a competing reaction to CO_2R. The possible reaction pathway can be illustrated as the following equations:

$$H^+ + e^- + * \rightarrow {}^*H \quad (15)$$

$$2^*H \rightarrow H_2 + 2* \quad (16)$$

To evaluate the selectivity and energy efficiency of CO_2R-to-C_1, it is necessary to investigate HER over different surfaces. Therefore, we have compared the free energy (ΔG_{HER}) on Cu (111), Sn (100), $Cu_{0.86}Sn_{0.14}$ (111), and Cu_6Sn_5 (−113) surfaces with varied concentrations of Sn. In this work, the calculations of HER are applied under standard conditions (P = 1 bar, T = 298.15 K) where the applied potential of 0 eV. ΔG_{HER} can be calculated as the following equation:[15]

$$\Delta G_{HER} = E_{total} - E_{slab} - \frac{1}{2}E_{H_2} + \Delta ZPE - T\Delta S_H \quad (17)$$

where E_{total} and E_{slab} can refer to equation (9). E_{H_2} denotes the ground energy of H_2 in the gas phase. ΔZPE is the zero-point energy correction of adsorbed hydrogen, which can be found in the equation (11). According to the reported literature, $\Delta ZPE - T\Delta S_H$ can be approximated as 0.24 eV.[15] In our theoretical work, the free energy of *H of 0 eV refers to the maximum HER. And a higher absolute value of ΔG_{HER} denotes decreased HER over the catalysts.

The possible adsorption configurations of *H on Cu (111), $Cu_{0.86}Sn_{0.14}$ (111), and Cu_6Sn_5 (−113) surfaces and their corresponding energetics can be observed in Supplementary Figs. 35−38, respectively.

Hybrid solvation and applied potential effects. Here, we applied the hybrid solvation effects using constant electrode potential (CEP) model via performing grand-canonical DFT calculations (GC-DFT). We compared the formation energy of the two crucial intermediates (*OCHO and *COOH) to represent the selectivity of CO_2R to formic acid vs. CO. To account for solvation effects, we employed both the implicit solvation model incorporated in VASPsol, along with explicit water molecules at the interface.[18-21] In the VASPsol model, the solvent was treated as a continuous medium, and its impact was represented using the dielectric constant of water. The electrolyte solution was modeled using a linearized Poisson-Boltzmann approach. Additionally, we implemented the Constant Electrode Potential (CEP) model to investigate surface charge and cation effects (e.g., K^+) on the CO_2R reaction through GC-DFT calculations.[22] At room temperature, the Debye screening length (Å) was calculated from the electrolyte concentration (M) using the following equation:[23]

$$\kappa = \frac{3}{\sqrt{I}} \tag{18}$$

In the equation, κ represents the Debye length, and I denotes the electrolyte concentration (e.g., 3 M K^+) in this part. In the CEP model, the applied potential was adjusted the Fermi level (E_f) towards a desired value by manipulating the number of electrons added or removed within the system. This adjustment by various number of the electrons can tune the work function (Φ) of the system and, thus, tune the applied potential (U_{SHE}) with a reference of the work function of Standard Hydrogen Electrode (Φ_{SHE}):

$$U_{SHE} = \frac{\Phi - \Phi_{SHE}}{e} \tag{19}$$

In the given context, Φ_{SHE} represents the work function of the standard hydrogen electrode, determined as Φ_{SHE}= 4.43 eV using RPBE for the thermodynamic work function of the standard hydrogen electrode.[24] This value serves as a reference for adjusting the number of additional electrons introduced into the systems. By altering the number of extra electrons added into the systems, we ensure a consistent potential of ~ −2.1 V vs. RHE for each examined elementary reaction, aligning with experimental

findings. The Gibbs reaction energy (G) under the constant electrode potential can be expressed as:[25]

$$G = E_{DFT} + ZPE - TS - \mu_e \times N_e - \mu_{cat/ani} \times |N_e| \tag{20}$$

where μ_e is the chemical potential of the electron. N_e is the number of electrons added or removed in the system. $\mu_{cat/ani}$ is the chemical potential of the cations/anions. Moreover, the *pH* correction to the Gibbs free energy can be given by:[16]

$$\Delta G_{pH} = -k_B T ln(|^{*}H|) = -0.0592\, pH \tag{21}$$

For the Gibbs free energy difference (ΔG) between *OCHO and *COOH over different surfaces investigated in this work, ΔG can be calculated as:

$$\Delta G = (E_{DFT\text{-}*COOH} - E_{DFT\text{-}*OCHO}) + (ZPE_{*COOH} - ZPE_{*OCHO}) - T(S_{*COOH} - S_{*OCHO}) - \mu_e \times (N_{e\text{-}*COOH} - N_{e\text{-}*OCHO}) - \mu_{cat/ani} \times (|N_{e\text{-}*COOH}| - |N_{e\text{-}*OCHO}|) - 0.0592\, pH \tag{22}$$

The divergence in Gibbs free energy between the two pivotal intermediates across the specified surfaces are shown in Fig. 2a in the manuscript, and the corresponding configurations are presented in Supplementary Fig. 48. In the presence of as solvation, applied potential, and cation effects, our conclusions remain the same that the formation energy difference between *COOH and *OCHO species as a volcano plot across diverse surfaces. The Cu_6Sn_5 (−113) surface exhibits the most significant distinction between these critical intermediates with a thermodynamic favorability of forming *OCHO (the intermediate for FA). In other words, the Cu_6Sn_5 (−113) facet will show the highest selectivity in the formic acid formation process as compared to other examined catalysts.

Supplementary Figures

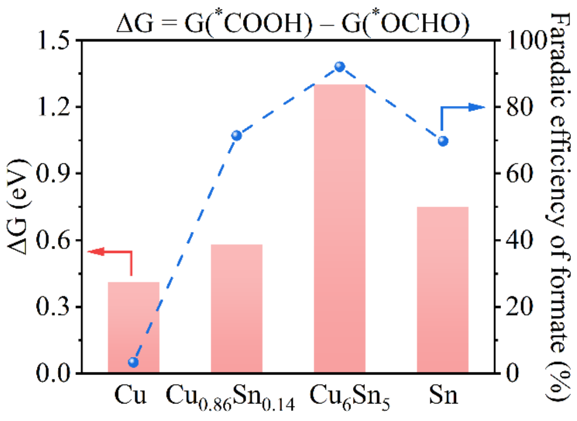

Supplementary Fig. 1 A volcano-like plot showing the Gibbs free energy difference (ΔG) between *OCHO (an intermediate for FA production) and *COOH (an intermediate for CO production) over Cu (111), $Cu_{0.86}Sn_{0.14}$ (111), Cu_6Sn_5 (−113), and Sn (100) surfaces. This plot is in line with the obtained experimental data of Faradaic efficiency (FE) for FA production at 1.2 A cm^{-2} in 1 M KOH. Gibbs free energy was calculated at room temperature (298.15 K), pH of 13.8, and an applied potential of −2.1 V vs. reversible hydrogen electrode (V_{RHE}).

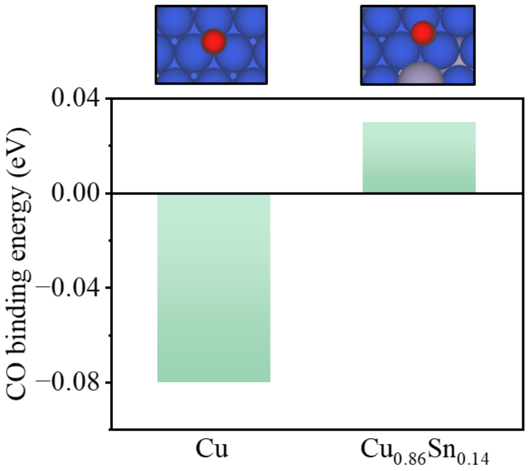

Supplementary Fig. 2 CO binding energy comparison over Cu (111) and $Cu_{0.86}Sn_{0.14}$ (111). The binding energy was calculated at room temperature (298.15 K).

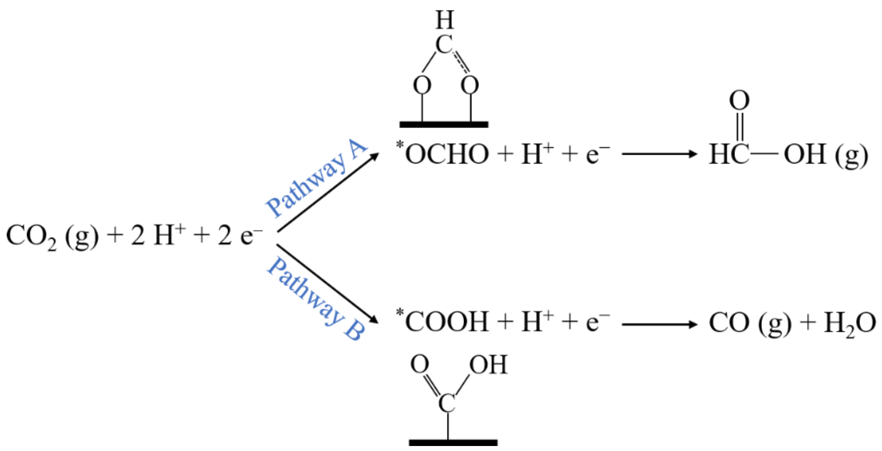

Supplementary Fig. 3 Possible reaction mechanisms of CO_2R to HCOOH and CO.

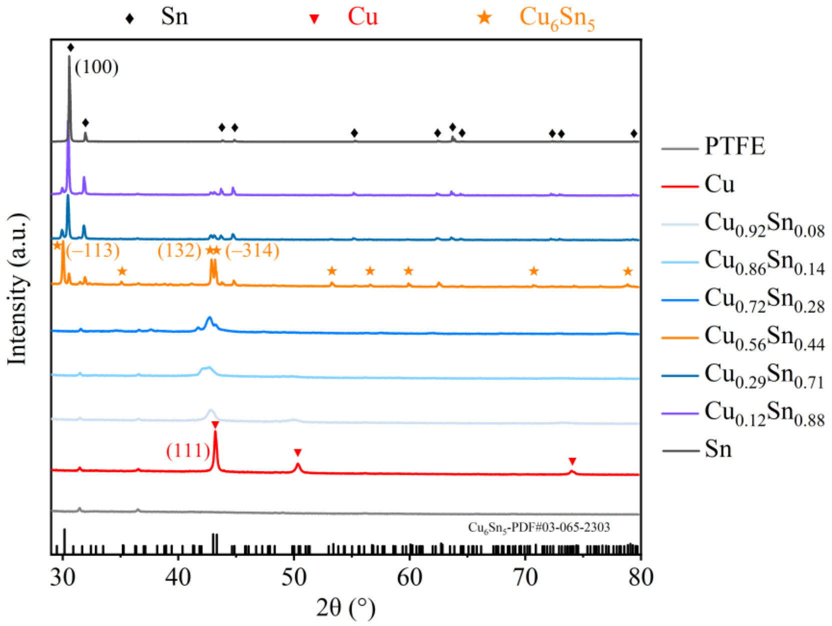

Supplementary Fig. 4 XRD of Cu, $Cu_{1-x}Sn_x$ ($x = 0.08, 0.14, 0.28, 0.44, 0.71, 0.88$) and Sn.

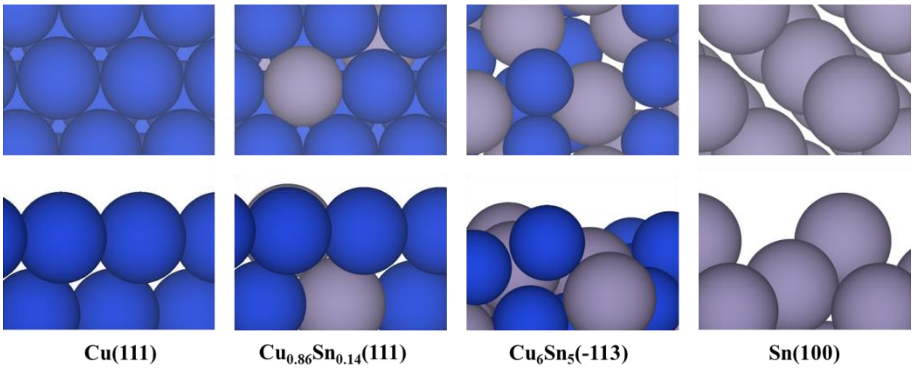

Supplementary Fig. 5 The top and side view of Cu (111), $Cu_{0.86}Sn_{0.14}$ (111), Cu_6Sn_5 (−113), and Sn (100). Color-coded atoms represent Cu (blue) and Sn (grey).

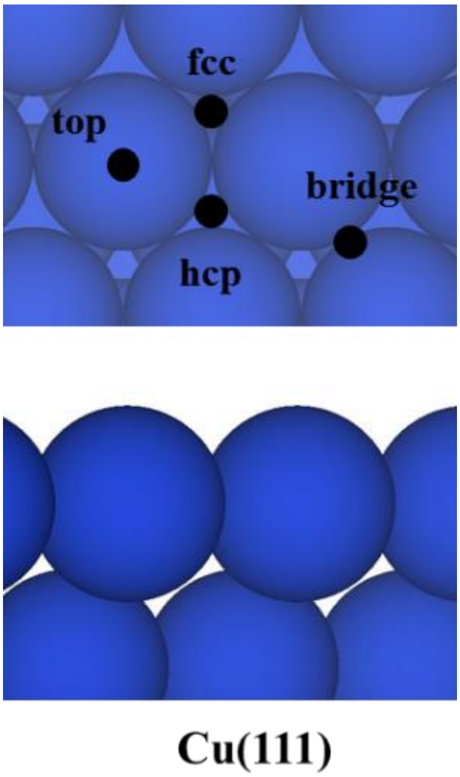

Supplementary Fig. 6 The possible adsorption sites over Cu (111).

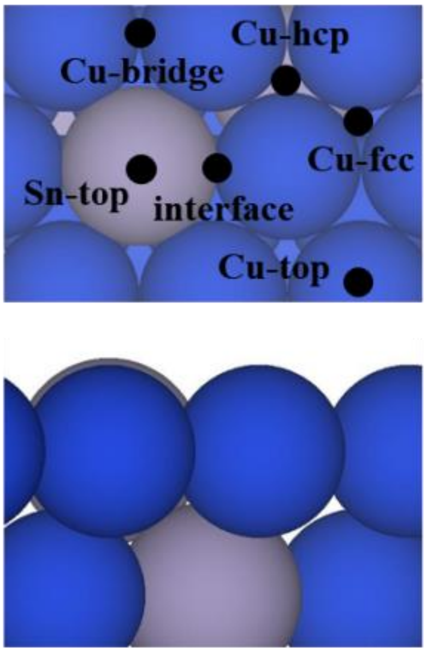

$Cu_{0.86}Sn_{0.14}$(111)

Supplementary Fig. 7 The possible adsorption sites over $Cu_{0.86}Sn_{0.14}$ (111).

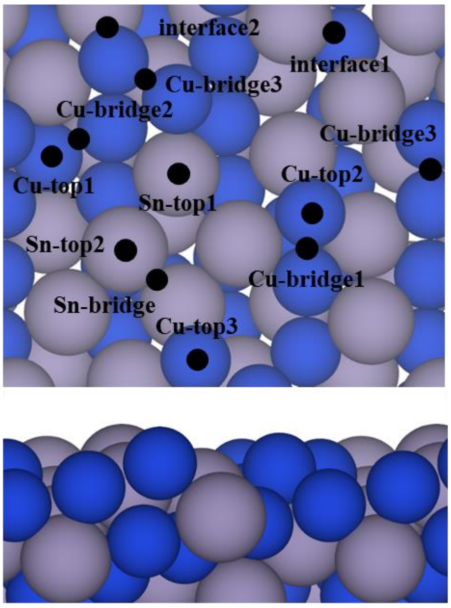

Cu_6Sn_5 (-113)

Supplementary Fig. 8 The possible adsorption sites over Cu_6Sn_5 (−113).

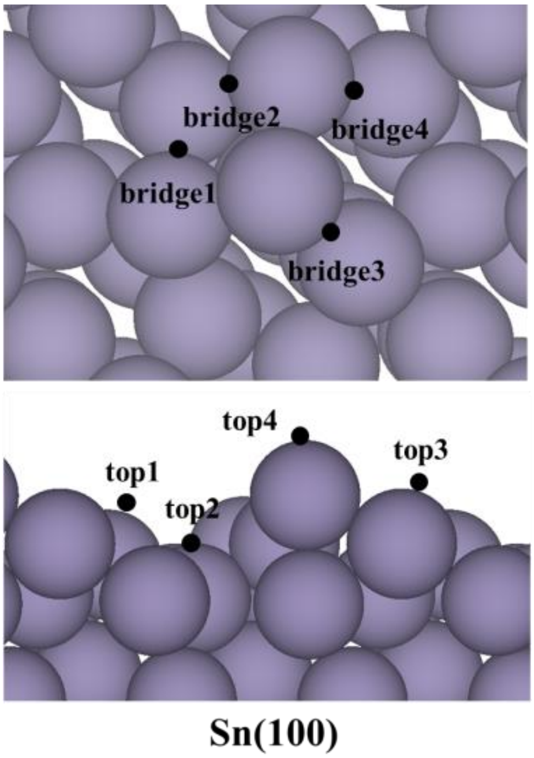

Supplementary Fig. 9 The possible adsorption sites over Sn (100) surface.

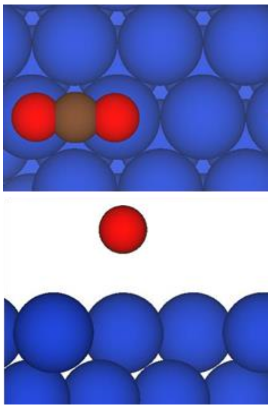

Supplementary Fig. 10 The possible adsorption configurations of CO_2 over a Cu (111) surface. CO_2 physically adsorbed over the Cu (111) surface with E_{ad} of 0 eV, calculated by equation (4).

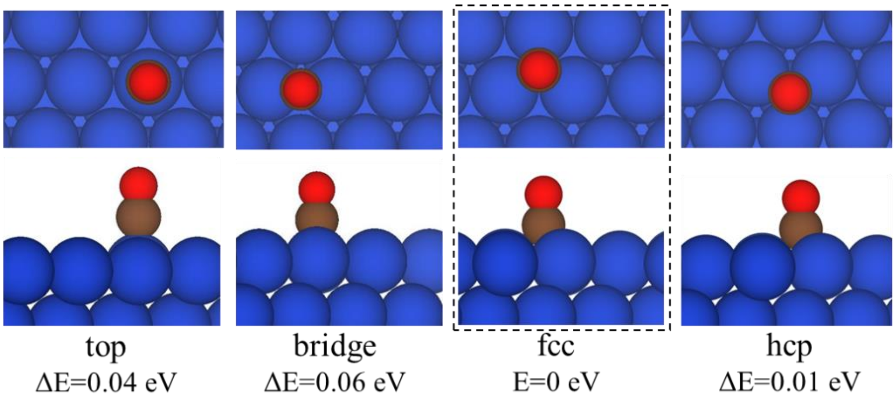

Supplementary Fig. 11 The possible adsorption configurations of *CO over a Cu (111) surface and their corresponding adsorption energies. The most favorable adsorption site of CO over Cu (111) is fcc with E_{ad} of −0.43 eV.

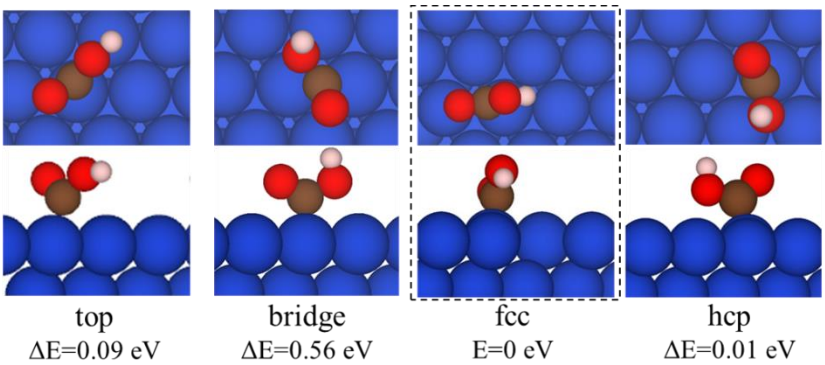

Supplementary Fig. 12 The possible adsorption configurations of key intermediate $^{*}COOH$ over a Cu (111) surface and their corresponding adsorption energies. The most favorable adsorption site of $^{*}COOH$ is the fcc site with E_{ad} of −1.16 eV.

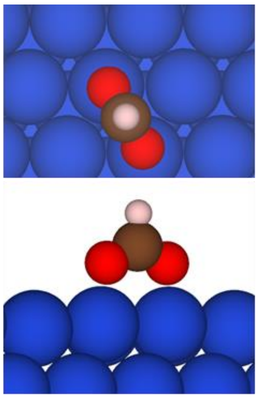

Supplementary Fig. 13 The possible adsorption configuration of key intermediate $^*OCHO$ over a Cu (111) surface and its corresponding adsorption energy. The most favorable adsorption site of $^*OCHO$ is the bridge site with E_{ad} of −1.85 eV. When $^*OCHO$ adsorbed over the top and hollow (fcc and hcp) sites, it is not stable, and shifts to the most favorable bridge site.

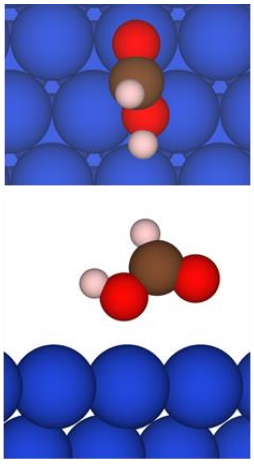

Supplementary Fig. 14 The possible adsorption configuration of FA over a Cu (111) surface. FA physically adsorbs over the surface with E_{ad} of −0.07 eV.

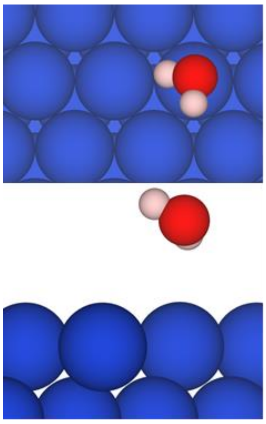

Supplementary Fig. 15 The possible adsorption configuration of H_2O over a Cu (111) surface. H_2O physically adsorbs over the surface with E_{ad} of 0.01 eV.

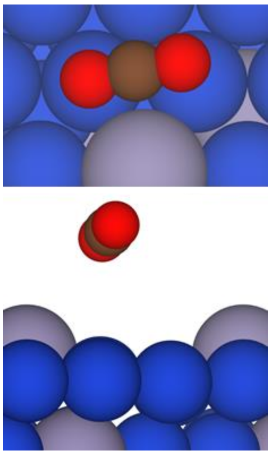

Supplementary Fig. 16 The possible adsorption configuration of CO_2 over a $Cu_{0.86}Sn_{0.14}$ (111) surface. CO_2 physically adsorbed over the surface with E_{ad} of 0.02 eV.

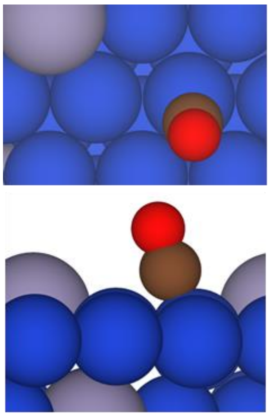

Supplementary Fig. 17 The possible adsorption configurations of *CO over a $Cu_{0.86}Sn_{0.14}$ (111) surface and its corresponding adsorption energy. The most favorable adsorption site of CO is the Cu-fcc site with E_{ad} of −0.36 eV. When CO is adsorbed over the interface and Sn top sites, it is not stable and shifts to the most favorable Cu-fcc site.

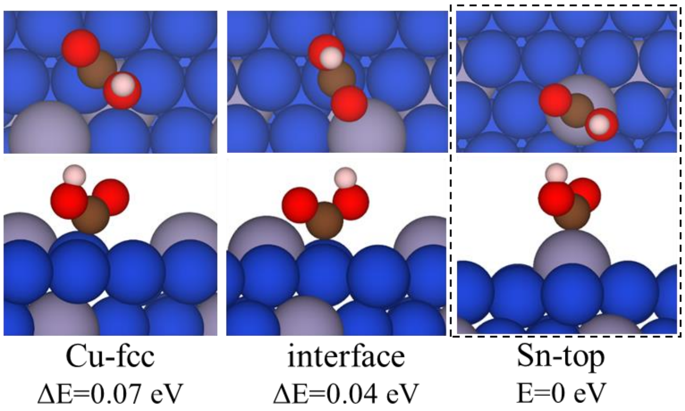

Supplementary Fig. 18 The possible adsorption configurations of key intermediate $^{*}COOH$ over a $Cu_{0.86}Sn_{0.14}$ (111) surface and their corresponding adsorption energies. The most favorable adsorption site of $^{*}COOH$ is the Sn top site with E_{ad} of −1.27 eV.

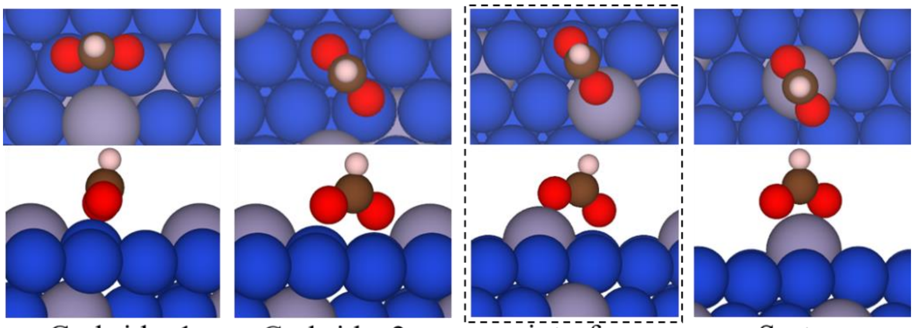

Supplementary Fig. 19 The possible adsorption configurations of key intermediate *OCHO over a $Cu_{0.86}Sn_{0.14}$ (111) surface and their corresponding adsorption energies. The most favorable adsorption site of *OCHO is the Sn-Cu interface bridge site with E_{ad} of −2.06 eV.

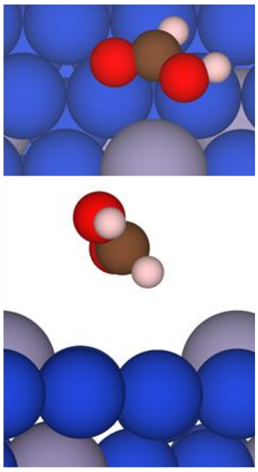

Supplementary Fig. 20 The possible adsorption configuration of FA over a $Cu_{0.86}Sn_{0.14}$ (111) surface. FA physically adsorbs over the surface with E_{ad} of −0.11 eV.

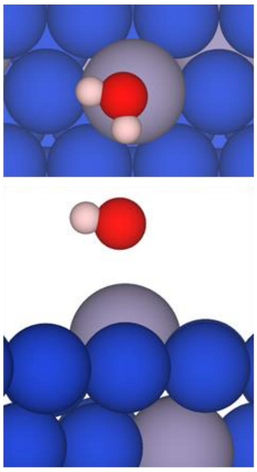

Supplementary Fig. 21 The possible adsorption configuration of H_2O over a $Cu_{0.86}Sn_{0.14}$ (111) surface. H_2O physically adsorbs over the surface with E_{ad} of 0 eV.

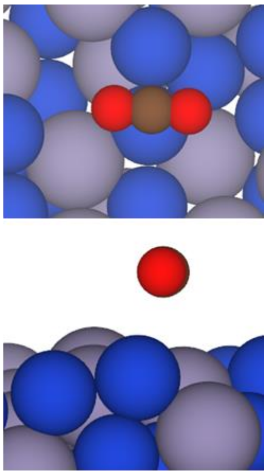

Supplementary Fig. 22 The possible adsorption configuration of CO_2 over a Cu_6Sn_5 (−113) surface. CO_2 physically adsorbs over the surface with E_{ad} of 0 eV.

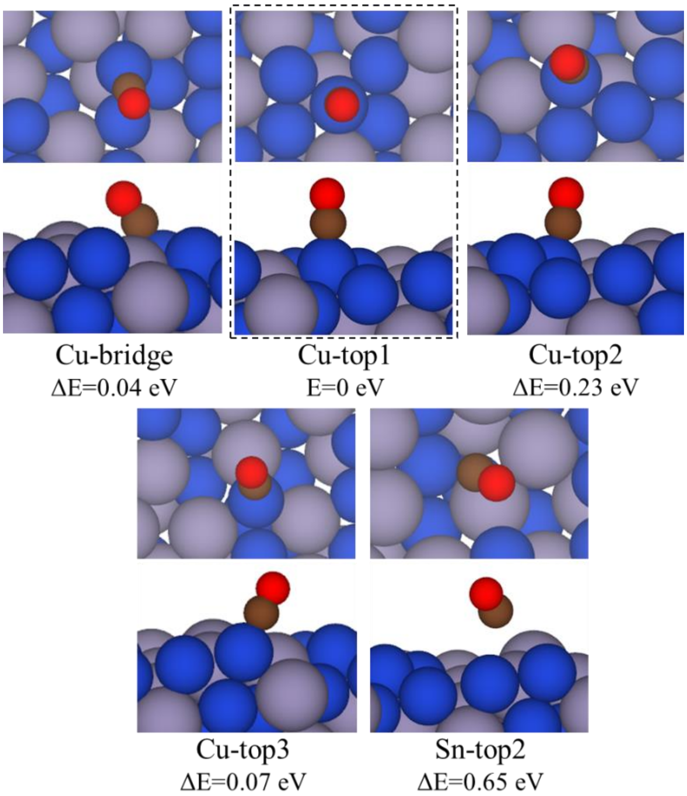

Supplementary Fig. 23 The possible adsorption configurations of $^{*}CO$ over a Cu_6Sn_5 (−113) surface and their corresponding adsorption energies. The most favorable adsorption site of $^{*}CO$ over the Cu_6Sn_5 (−113) surface is the Cu-top1 site with E_{ad} of −0.93 eV. When CO is adsorbed over the bridge site of two Sn atoms or other interface bridge sites between Cu and Sn atoms, it is not stable and shifts to the top site of Sn and the top site of Cu.

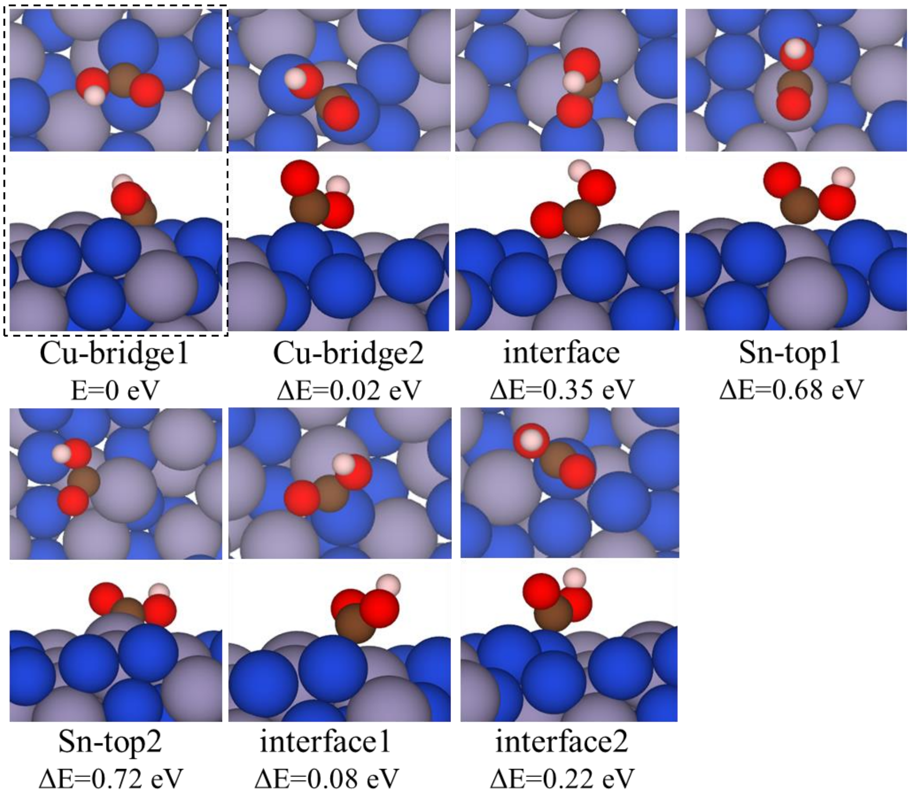

Supplementary Fig. 24 The possible adsorption configurations of $^{*}COOH$ over a Cu_6Sn_5 (−113) surface and their corresponding adsorption energies. The most favorable adsorption site of $^{*}COOH$ over the Cu_6Sn_5 (−113) surface is the Cu-bridge1 site with E_{ad} of −1.94 eV. When $^{*}COOH$ adsorbed over the Sn-bridge site or other interface sites between Cu and Sn atoms, it is not stable, and shifts to the Sn-top1 site, the interface1, and interface2 sites.

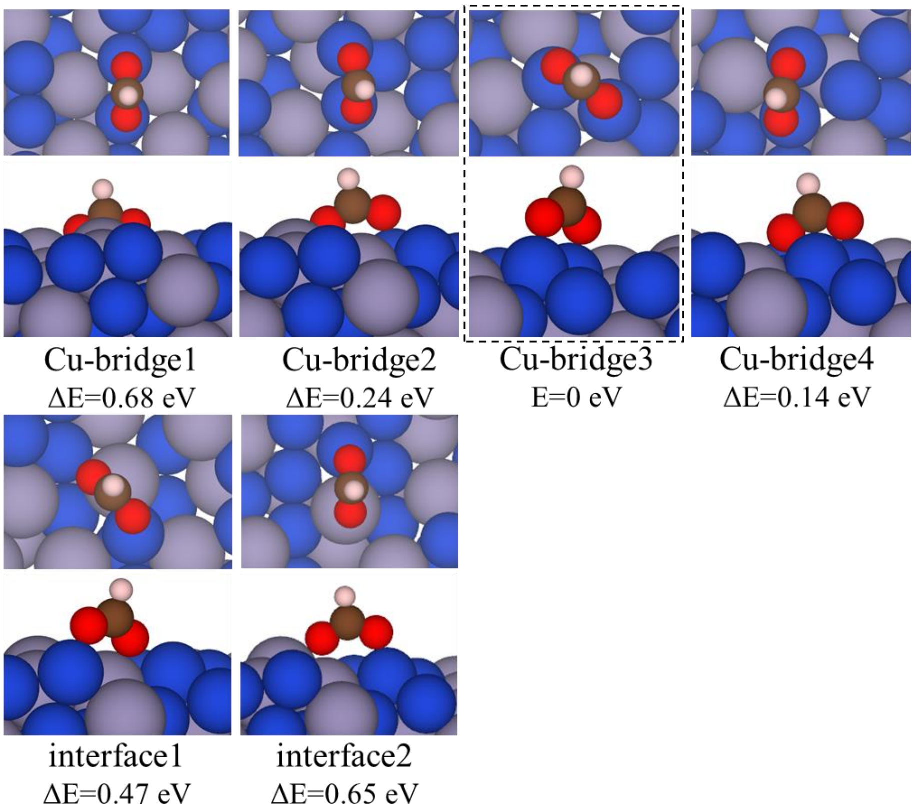

Supplementary Fig. 25 The possible adsorption configurations of *OCHO over a Cu_6Sn_5 (−113) surface and their corresponding adsorption energies. The most favorable adsorption site of *OCHO over the Cu_6Sn_5 (−113) surface is the Cu-bridge3 site with E_{ad} of −3.01 eV. When *COOH adsorbed over the top and bridge sites over Sn atoms or other interface sites between Cu and Sn atoms, it is not stable, and shifts to the interface1 and interface2 sites.

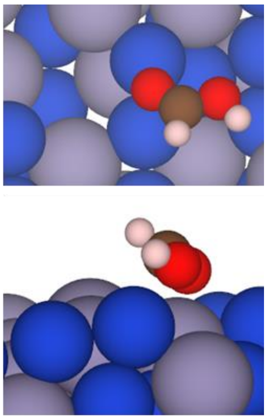

Supplementary Fig. 26 The possible adsorption configuration of FA over a Cu_6Sn_5 (−113) surface. FA physically adsorbs over the surface with E_{ad} of −0.29 eV.

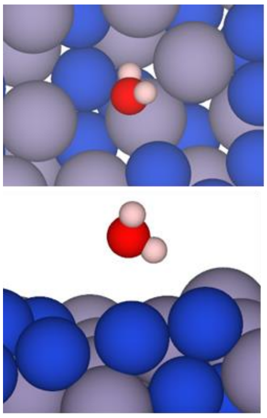

Supplementary Fig. 27 The possible adsorption configuration of H_2O over a Cu_6Sn_5 (−113) surface. H_2O physically adsorbs over the surface with E_{ad} of 0.02 eV.

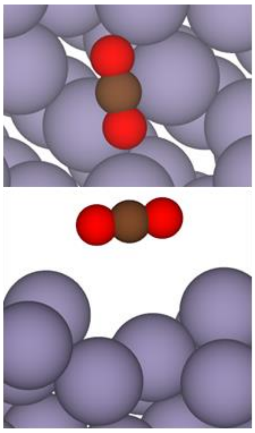

Supplementary Fig. 28 The possible adsorption configurations of CO_2 over a Sn (100) surface. CO_2 physically adsorbs over the surface with E_{ad} of 0 eV.

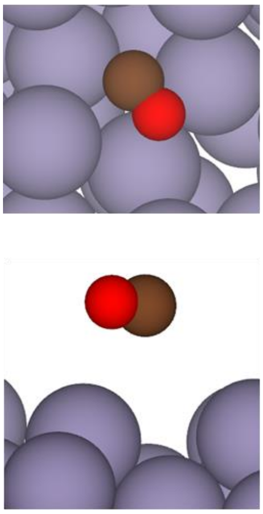

Supplementary Fig. 29 The possible adsorption configuration of $^{*}CO$ over a Sn (100) surface. CO physically adsorbs over the surface with E_{ad} of −0.01 eV.

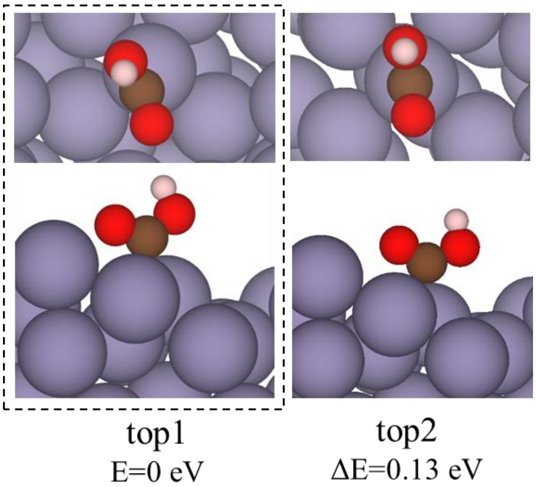

Supplementary Fig. 30 The possible adsorption configurations of key intermediate *COOH over a Sn (100) surface and their corresponding adsorption energies. The most favorable adsorption site for *COOH over the Sn (100) surface is the top site of the upper Sn atom with E_{ad} of −1.34 eV. When *COOH adsorbed over the bridge sites and other top sites among Sn atoms, it is not stable, and shifts to the top1 and top2 sites.

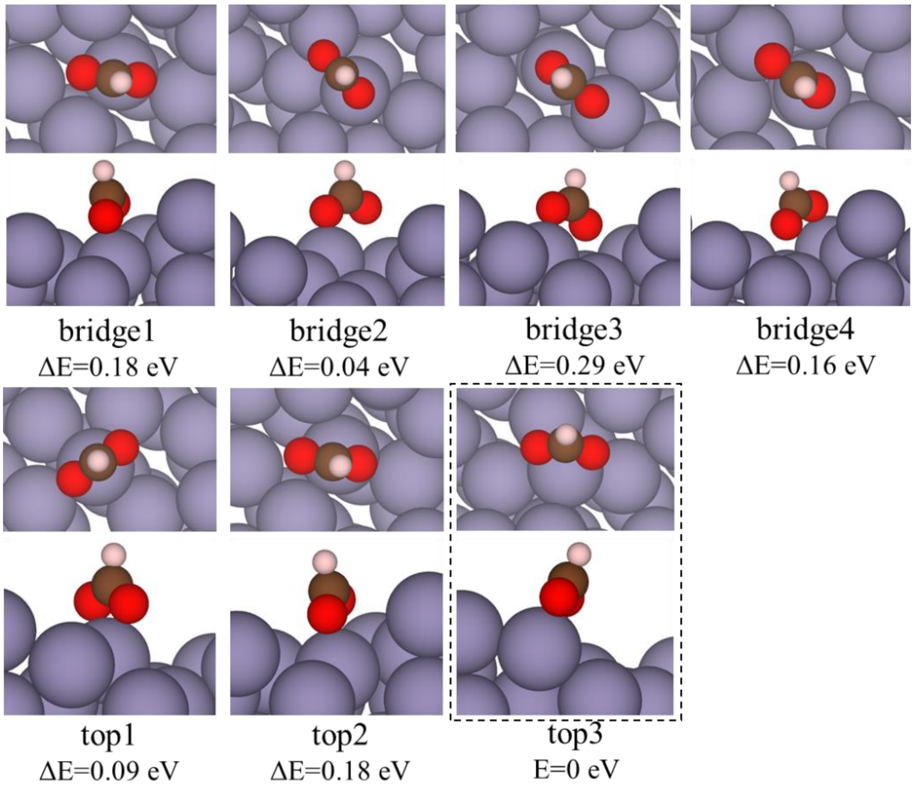

Supplementary Fig. 31 The possible adsorption configurations of key intermediate *OCHO over a Sn (100) surface and their corresponding adsorption energies. The most favorable adsorption site of *OCHO over the Sn (100) surface is the top3 site with E_{ad} of −2.33 eV. When *OCHO adsorbed over the top4 sites over Sn (100), it is not stable, and shifts to the most favorable top3 site.

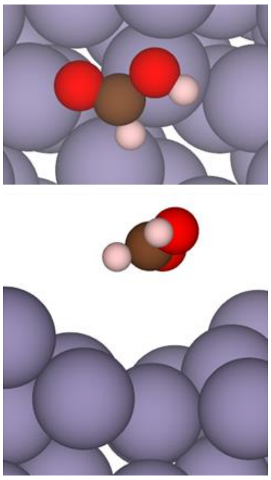

Supplementary Fig. 32 The possible adsorption configurations of FA over a Sn (100) surface. FA physically adsorbs over the surface with E_{ad} of −0.02 eV.

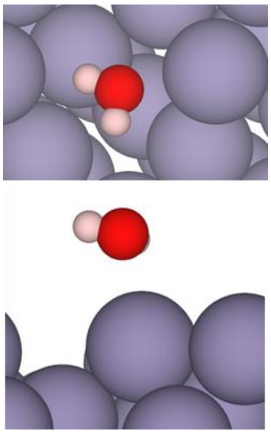

Supplementary Fig. 33 The possible adsorption configuration of H_2O over a Sn (100) surface. H_2O physically adsorbs over the surface with E_{ad} of 0.05 eV.

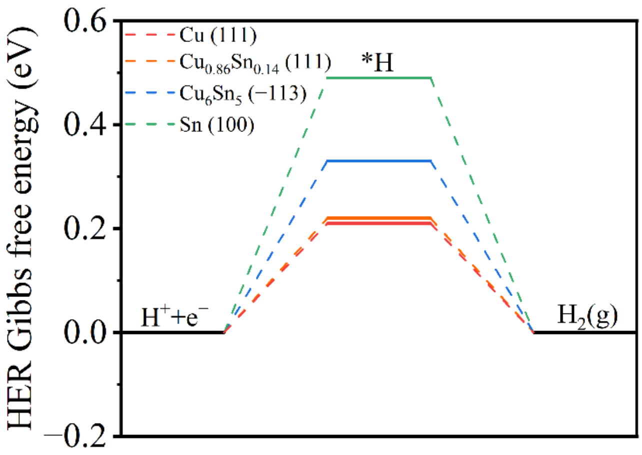

Supplementary Fig. 34 Gibbs free energy diagrams of hydrogen evolution reaction (HER) on Cu (111), $Cu_{0.86}Sn_{0.14}$ (111), Cu_6Sn_5 (−113) and Sn (100) surfaces, respectively.

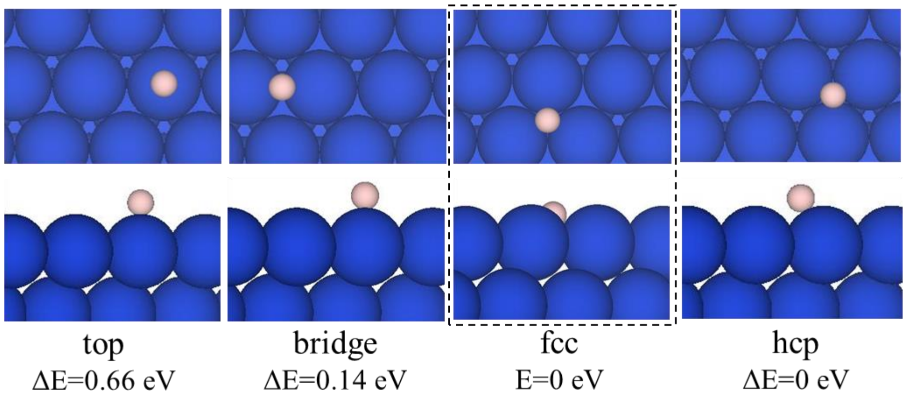

Supplementary Fig. 35 The possible adsorption configurations of *H over a Cu (111) surface and their corresponding adsorption energies. The most favorable adsorption site of *H over Cu (111) is fcc with E_{ad} of −0.04 eV.

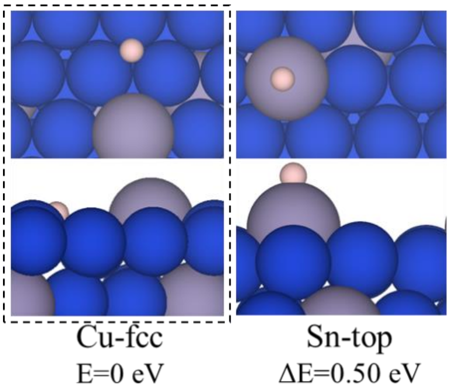

Supplementary Fig. 36 The possible adsorption configurations of $^*H$ over a $Cu_{0.86}Sn_{0.14}$ (111) surface and their corresponding adsorption energies. The most favorable adsorption site of $^*H$ over $Cu_{0.86}Sn_{0.14}$ (111) is Cu-fcc with E_{ad} of 0.05 eV. When hydrogen is adsorbed over the interface sites, it is not stable and shifts to the most favorable Cu-fcc site.

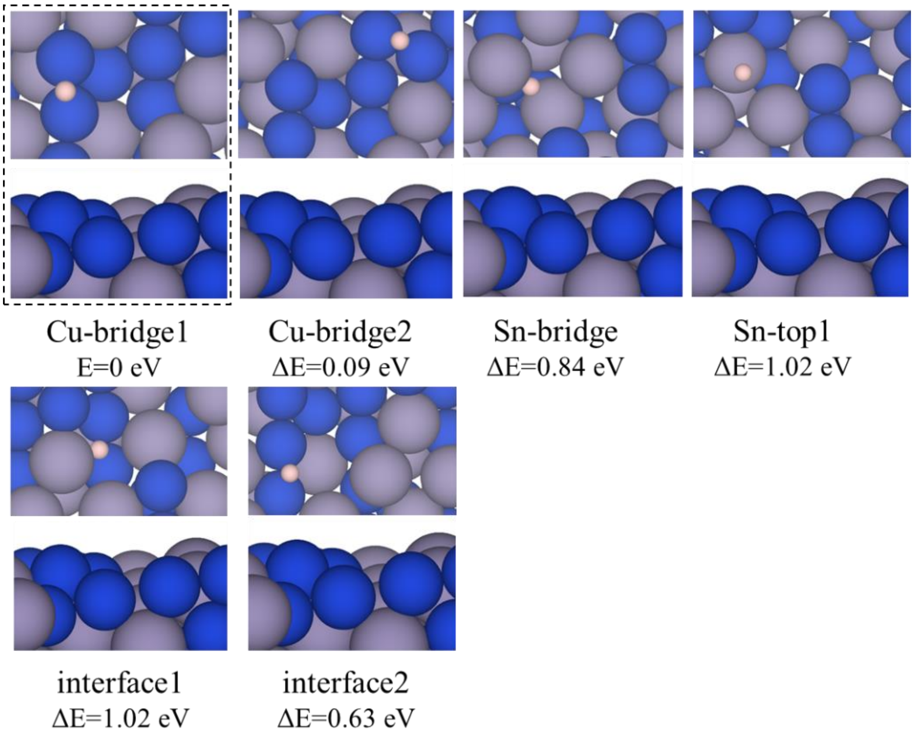

Supplementary Fig. 37 The possible adsorption configurations of $^*H$ over a Cu_6Sn_5 (−113) surface and their corresponding adsorption energies. The most favorable adsorption site of $^*H$ over Cu_6Sn_5 (−113) is Cu-bridge1 with E_{ad} of −0.25 eV. When hydrogen is adsorbed over the Cu-top and Sn-top2 sites, it is not stable, and shifts to the Cu-bridge2 and Cu-bridge1 sites, respectively.

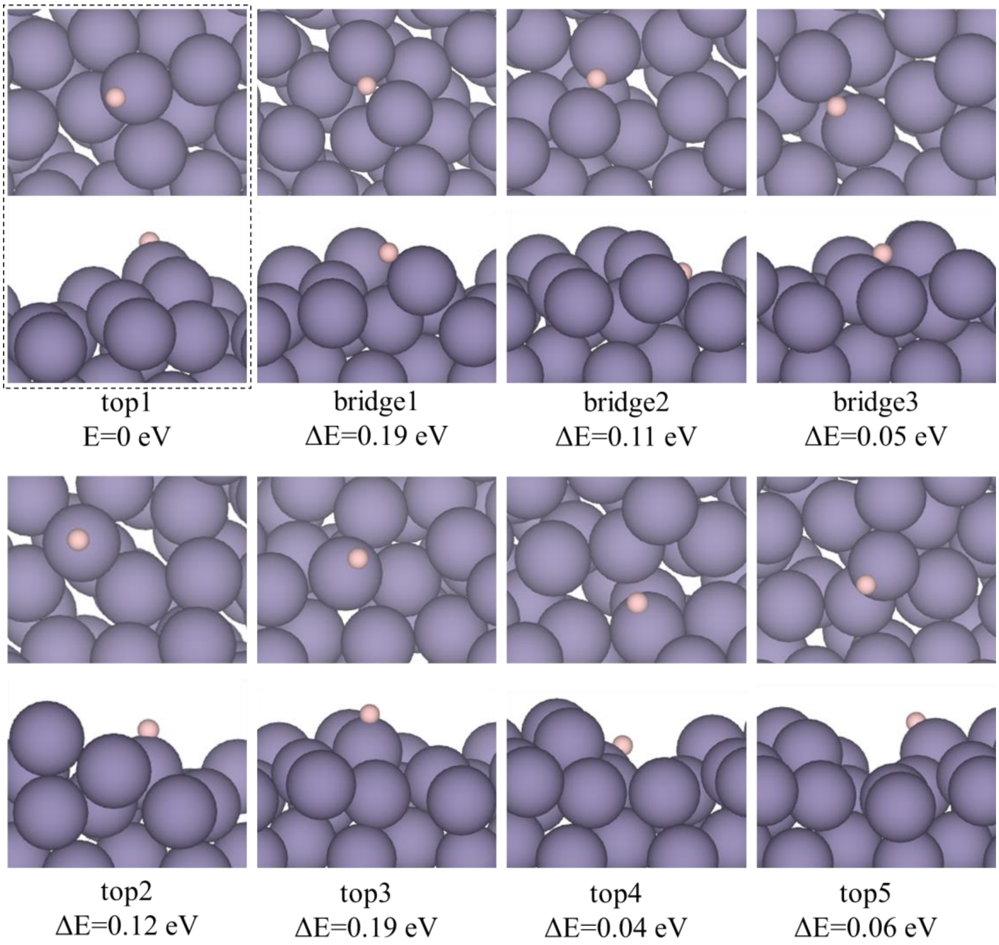

Supplementary Fig. 38 The possible adsorption arrangements of $^{*}H$ on a Sn (100) surface and their associated adsorption energies are considered. The preferred adsorption site for $^{*}H$ on Sn (100) is determined to be the top site, with an adsorption energy (E_{ad}) of 0.25 eV.

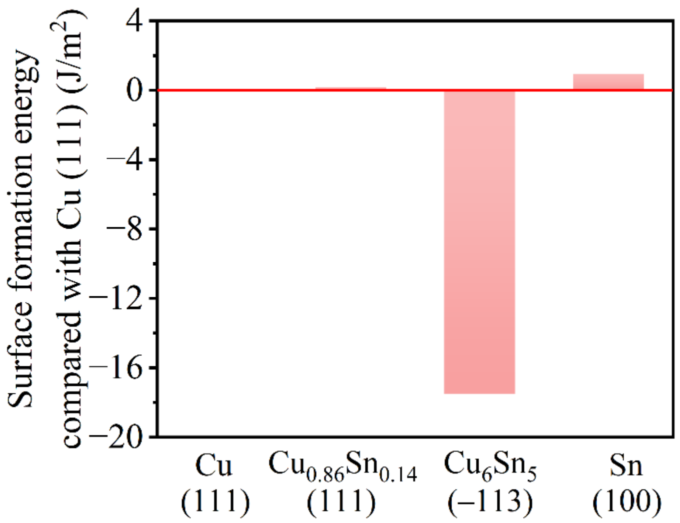

Supplementary Fig. 39 DFT calculated surface formation energies for $Cu_{0.86}Sn_{0.14}$ (111), Cu_6Sn_5 (−113), and Sn (100) surfaces with a reference of the ones for Cu (111) of ~0.98 J/m^2.

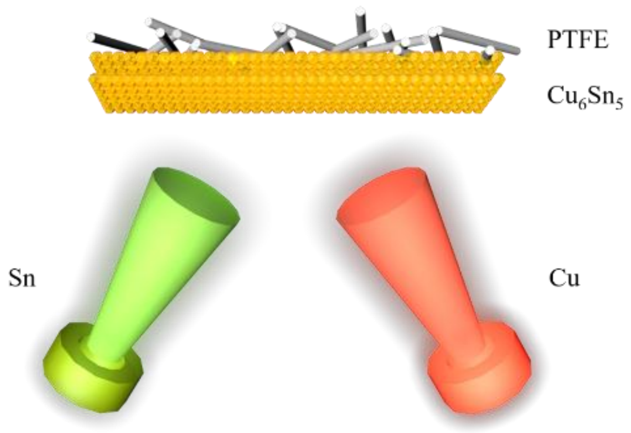

Supplementary Fig. 40 Schematic illustration of the synthetic process of $Cu_{1-x}Sn_x$ (x = 0.08, 0.14, 0.28, 0.44, 0.71, 0.88) catalysts on polytetrafluoroethylene (PTFE) gas diffusion electrodes.

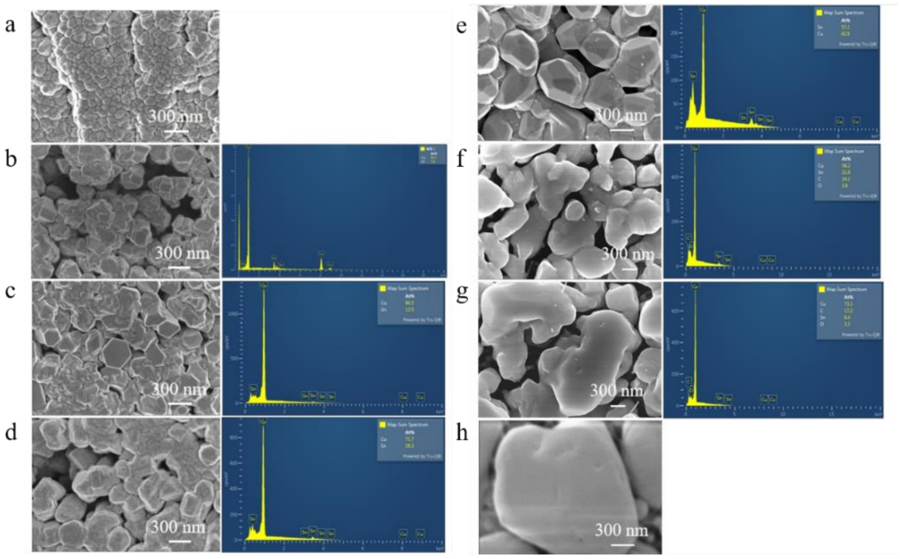

Supplementary Fig. 41 SEM and EDX spectrum of Cu (a), $Cu_{0.92}Sn_{0.08}$ (b), $Cu_{0.86}Sn_{0.14}$ (c), $Cu_{0.72}Sn_{0.28}$ (d), $Cu_{0.56}Sn_{0.44}$ (e), $Cu_{0.29}Sn_{0.71}$ (f), $Cu_{0.12}Sn_{0.88}$ (g), and Sn (h).

The $Cu_{1-x}Sn_x$ particles formed through evaporation exhibited an increase in size corresponding to higher Sn ratios. Notably, we successfully achieved the formation of Cu_6Sn_5 particles with diameters ranging from 300−600 nm over a large area on PTFE, when adjusting the Cu: Sn molar ratio to approximately 56:44.

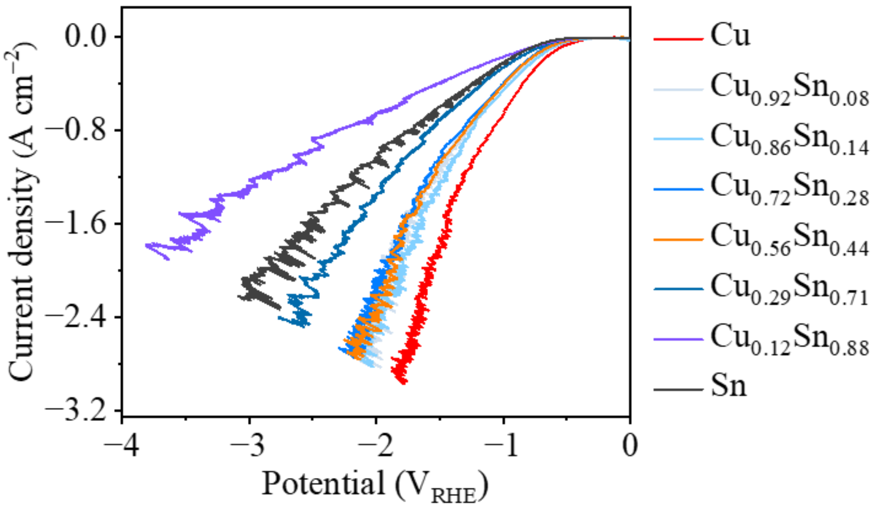

Supplementary Fig. 42 LSV curves of Cu, $Cu_{1-x}Sn_x$ (x = 0.08, 0.14, 0.28, 0.44, 0.71, 0.88), and Sn in 1 M KOH for CO_2 reduction (with *iR* compensation).

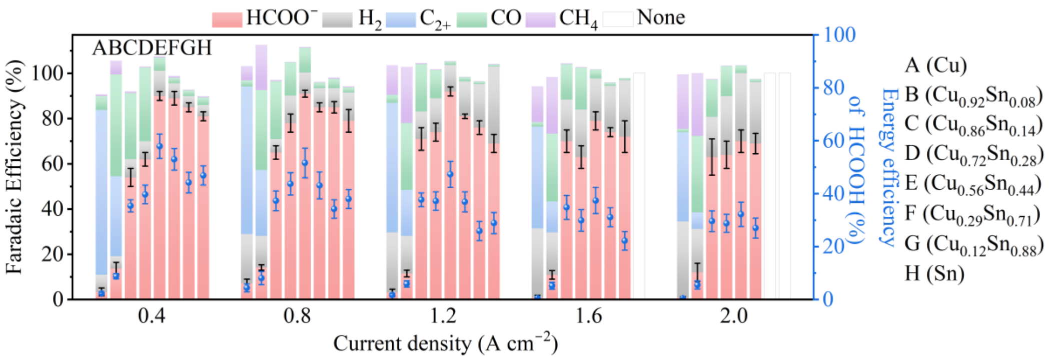

Supplementary Fig. 43 Faradaic efficiencies (FEs) and energy efficiencies (EEs) for Cu, $Cu_{1-x}Sn_x$ (x = 0.08, 0.14, 0.28, 0.44, 0.71, 0.88) and Sn catalysts at various current densities of 0.4, 0.8, 1.2, 1.6, and 2.0 A cm^{-2} in 1 M KOH at pH = 14 in flow cells. The error bars presented are derived from three independent tests.

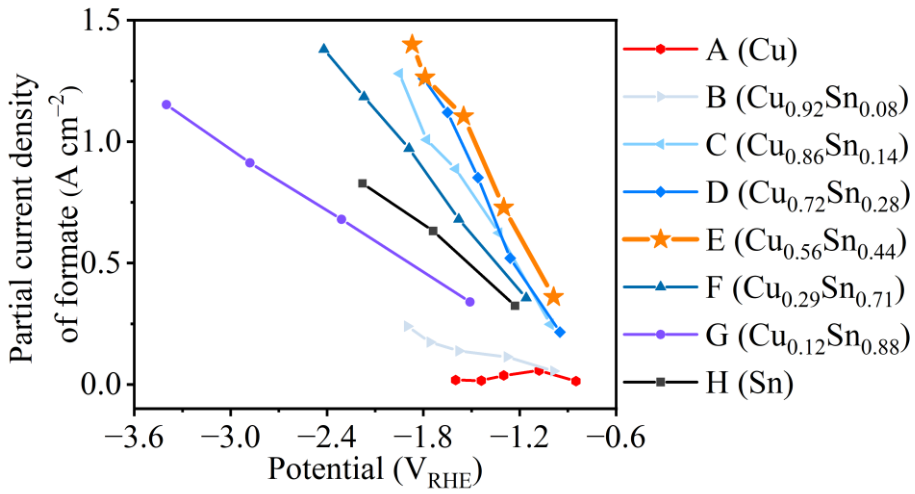

Supplementary Fig. 44 Partial current densities of $HCOO^{-}$ as a function of the applied potentials on Cu, $Cu_{1-x}Sn_x$ (x = 0.08, 0.14, 0.28, 0.44, 0.71, 0.88), and Sn catalysts in 1 M KOH at pH = 14 in flow cells.

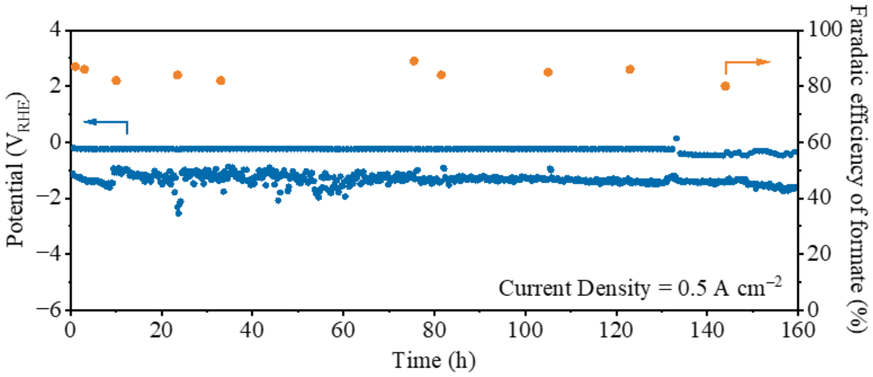

Supplementary Fig. 45 The 160-hour stability test of Cu_6Sn_5 in 1 M KOH at 0.5 A cm^{-2} (with *iR* compensation).

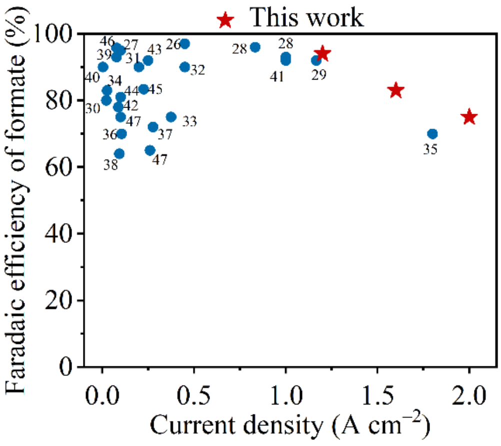

Supplementary Fig. 46 The comparison of this work with previously published data under alkaline conditions.[26–47]

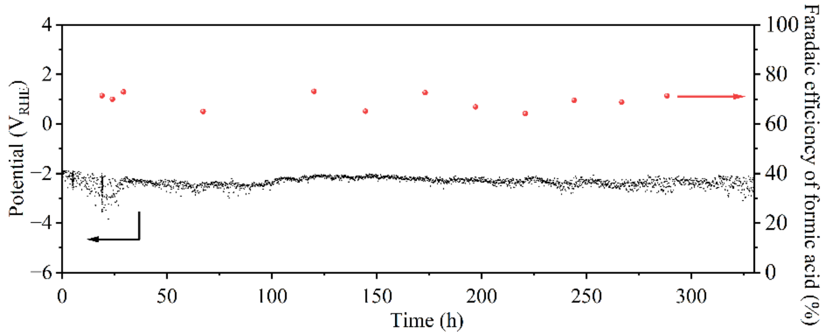

Supplementary Fig. 47 The 300-hour stability test of Cu_6Sn_5 in 3 M KCl and 0.05 M H_2SO_4 at 500 mA cm^{-2} at pH 1 in a flow cell (with *iR* compensation).

- In the case of alkaline CO_2R, we employed an alternating current density mode (0.05 A cm^{-2} for 30 s and 0.5 A cm^{-2} for 90 s) to assess CO_2R performance. This alternative current density mode, as previously reported in (*ACS Energy Lett.* 2021, **6**, (2), 809–815; *ACS Catal.* 2020, **10**, 12403–12413)[48,49], mitigates carbonate precipitation during alkaline CO_2R, ensuring the stable diffusion of CO_2 for continuous CO_2R.
- In the case of acidic CO_2R, the use of strong acid electrolytes mitigates severe carbonate precipitation. Therefore, we adopted normal chronopotentiometry to evaluate CO_2R performance under these conditions.

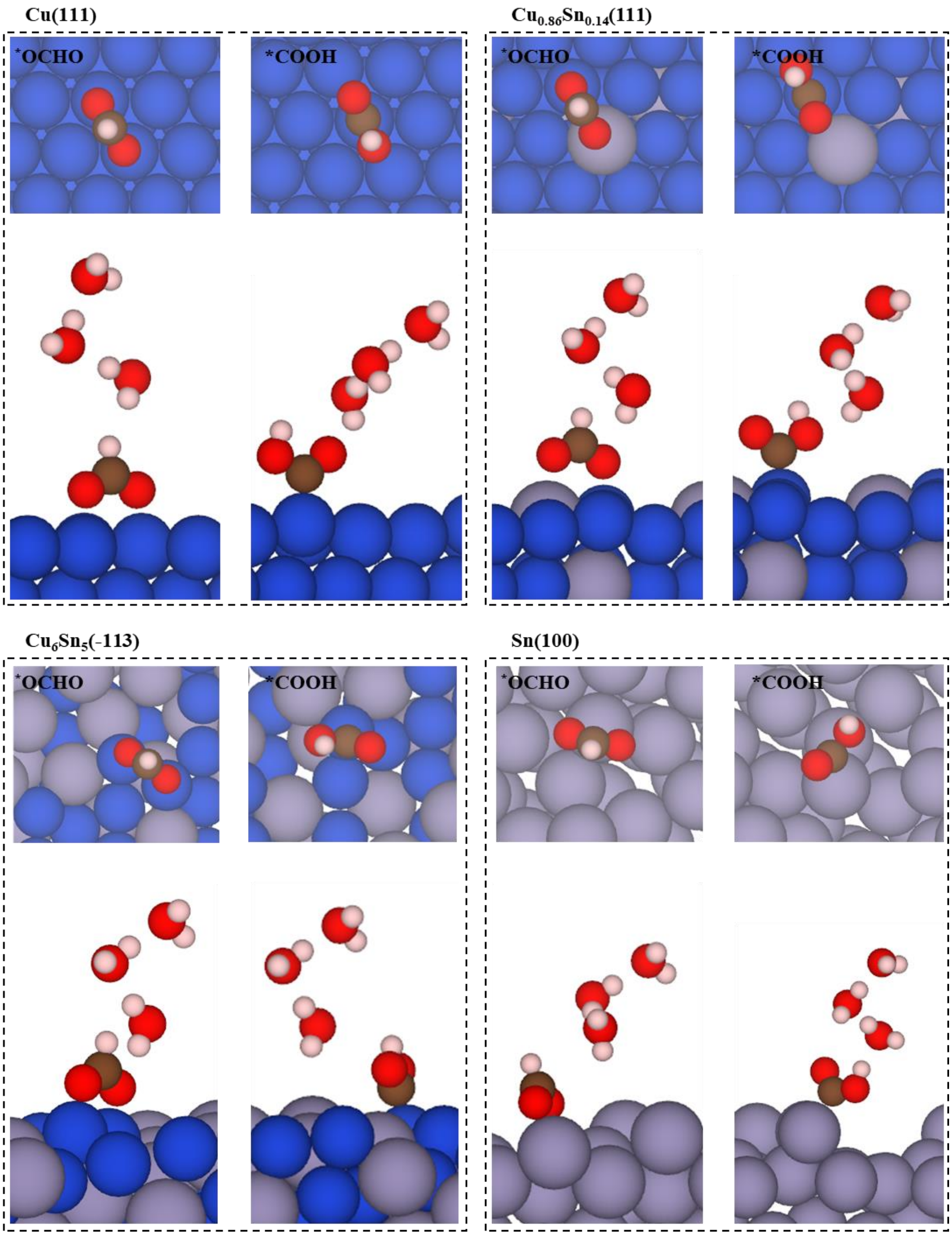

Supplementary Fig. 48 The adsorption configurations of two key intermediates (*OCHO and *COOH) on Cu (111), $Cu_{0.86}Sn_{0.14}$ (111), Cu_6Sn_5 (−113), and Sn (100) surfaces with considering hybrid solvation effects and utilizing CEP model with an applied potential of −2.1 V_{RHE}.

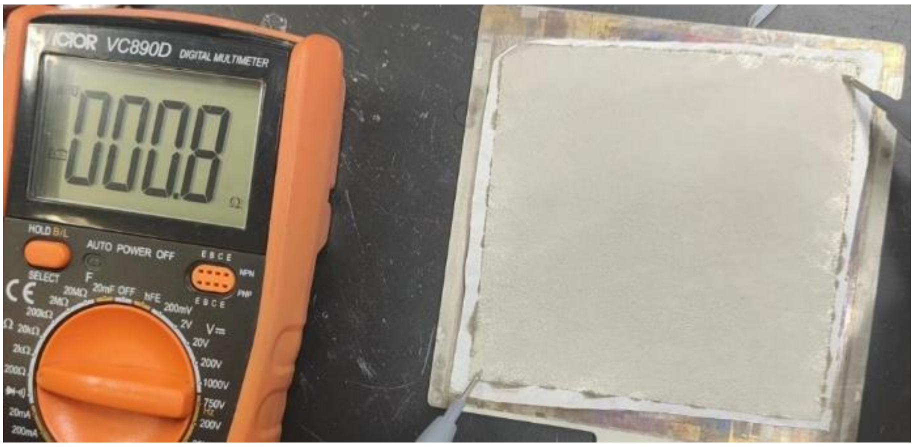

Supplementary Fig. 49 The resistance between two points of the as-prepared Cu_6Sn_5 catalyst.

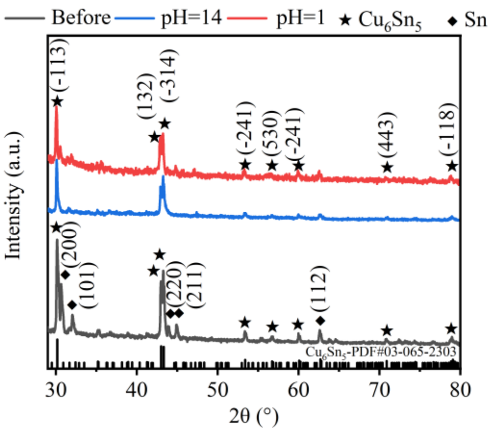

Supplementary Fig. 50 XRD comparison before and after 120-hour stability test in 1 M KOH or 3 M KCl and 0.05 M H_2SO_4 at 0.5 A cm^{-2}.

The excessive Sn was mostly removed after 5−10 hours of CO_2R reaction in alkaline (1 M KOH) or acid (3 M KCl and 0.05 M H_2SO_4) electrolytes, as confirmed by the post-reaction XRD analyses (Supplementary Fig. 50).

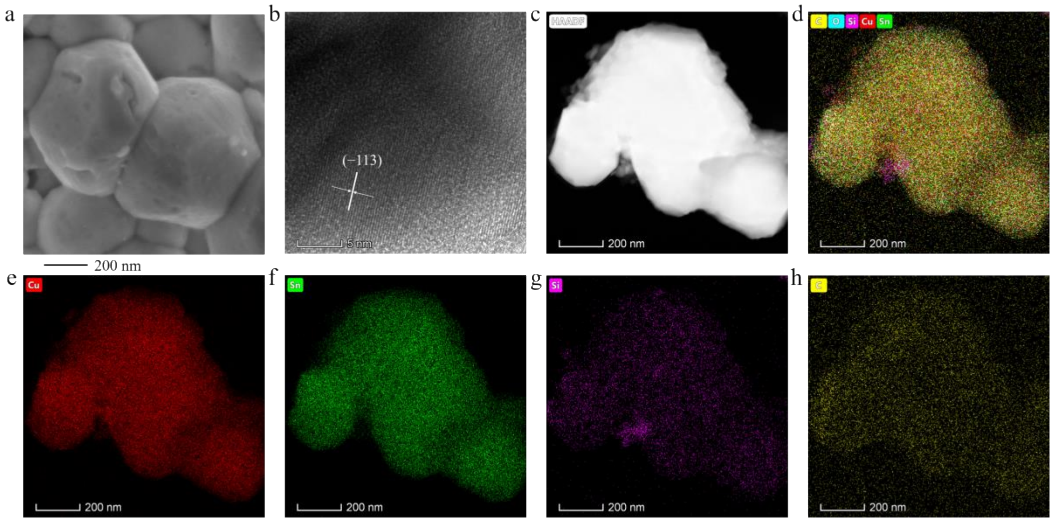

Supplementary Fig. 51 (a) Scanning electron microscopy (SEM), (b) high-resolution transmission electron microscopy (HRTEM), and (c-h) scanning transmission electron microscopy with energy dispersive X-ray spectroscopy (STEM-EDX) images of Cu_6Sn_5 following the 120-hour stability test conducted at 0.5 A cm^{-2} at pH 1.

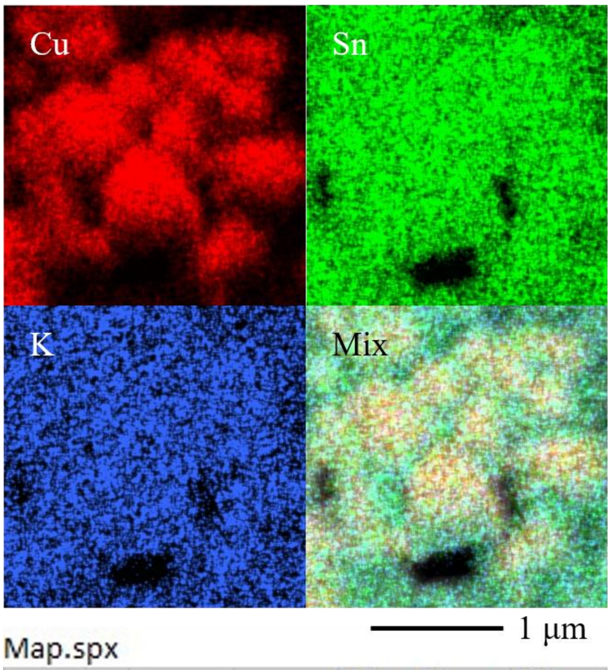

Map.spx

Element	At. No.	Line s.	Mass Norm. [%]	Atom [%]
Cu	29	L-Serie	37.07	52.39
Sn	50	L-Serie	62.93	47.61
			**100.00**	**100.00**

Supplementary Fig. 52 Scanning electron microscopy with energy dispersive X-ray spectroscopy (SEM-EDX) images of Cu_6Sn_5 following the 120-hour stability test conducted at 0.5 A cm^{-2} at pH 1.

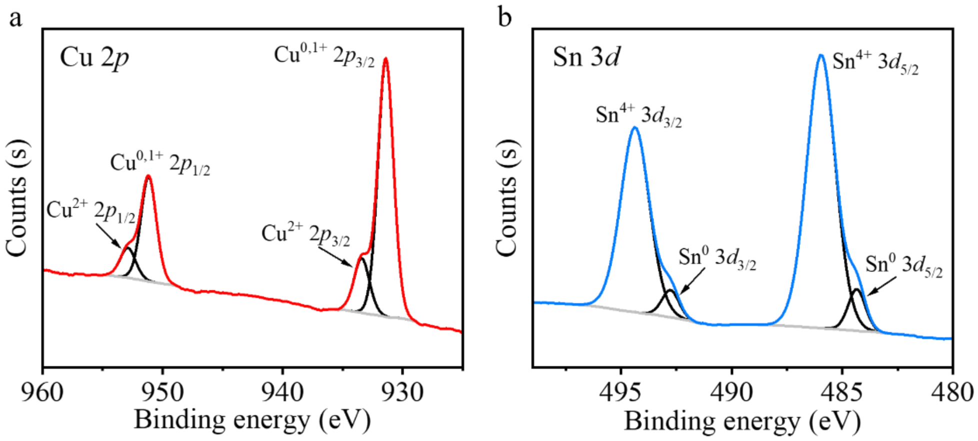

Supplementary Fig. 53 XPS spectra of Cu_6Sn_5 catalyst ((a) Cu 2*p*; (b) Sn 3*d*) after the 120-hour stability test under strong acid conditions.

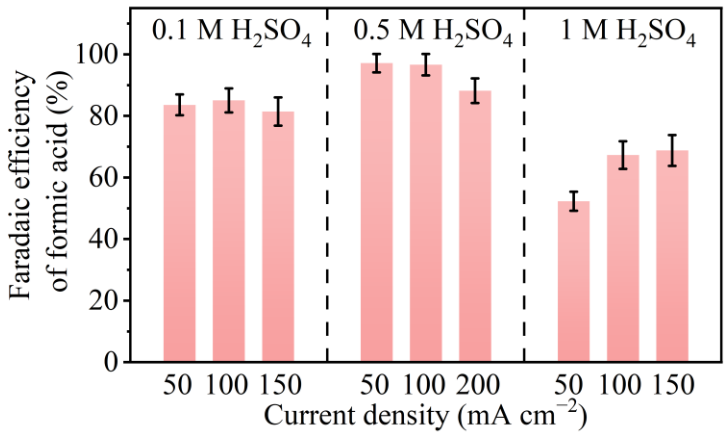

Supplementary Fig. 54 The CO_2R performance of Cu_6Sn_5 in different anolytes of 0.1, 0.5 and 1 M H_2SO_4 at different applied current densities in an SSE-based MEA.

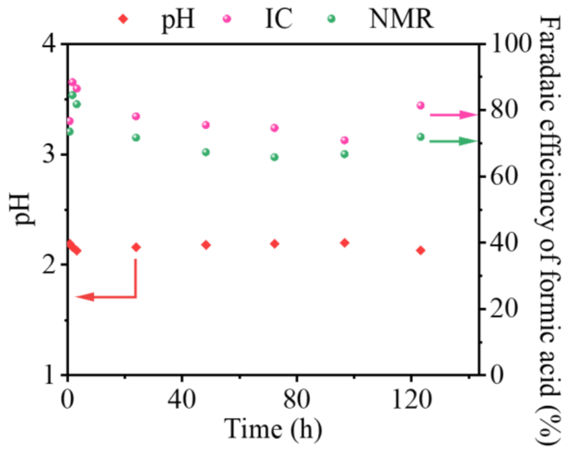

Supplementary Fig. 55 The recorded pH of the produced FA solution during the 130-h CO_2R stability test, along with the measured FE of FA by ion chromatography and nuclear magnetic resonance spectroscopy during the test.

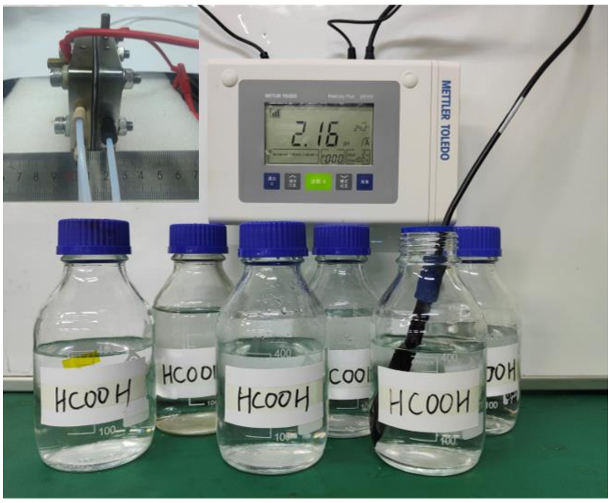

Supplementary Fig. 56 Optical image of the generated 2.6 liters of 0.36 M pure FA solution.

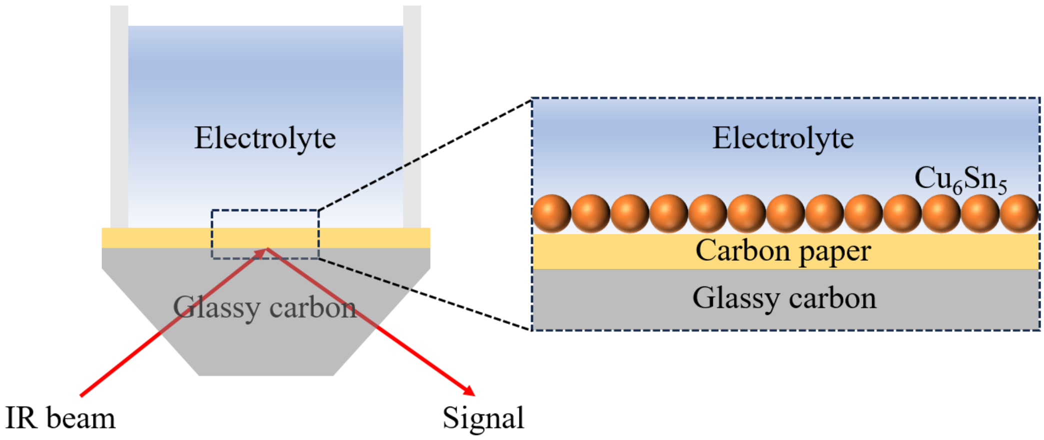

Supplementary Fig. 57 Schematic of *in situ* ATR-FTIR electrochemical measurement.

Supplementary Table 1. The adsorption energies (E_{ad}/eV) of various species on different catalysts.

	Cu (111)	$Cu_{0.86}Sn_{0.14}$ (111)	Cu_6Sn_5 (−113)	Sn (100)
CO_2	0	0.02	0	0
$^*CO$	−0.43	−0.36	−0.93	−0.01
$^*COOH$	−1.16	−1.27	−1.94	−1.34
$^*OCHO$	−1.85	−2.06	−3.01	−2.33
FA	−0.07	−0.11	−0.29	−0.02
H_2O	0.01	0	0.02	0.05
$^*H$	−0.04	0.05	−0.25	

Supplementary Table 2. Comparison of our work with previously published data under both acidic and alkaline conditions.

Cat.	electrolyte	FE (%)	EE (%)	Stability (h)	Ref.
Cu_6Sn_5	3 M KCl +0.05 M H_2SO_4	96	52.2	300	This work
Cu_6Sn_5	1 M KOH	92	57.9	120	This work
Cu_6Sn_5	MEA	88	37	130	This work
nBuLi-Bi	MEA	~96	–	100	26
2D-Bi	MEA	>90	48.5/43.3	100	27
InP CQDs	1 M KOH	93	–	4	28
Pb_1Cu	MEA	85	35.2	180	29
Sn quantum sheets	0.1 M $NaHCO_3$	89	–	50	30
BiOBr-templated catalyst	1 M $KHCO_3$	>90	–	65	31
carbon-supported SnO_2	MEA	90	15.3	11	32
carbon-supported SnO_2	0.1 M $KHCO_3$/ 1 M KOH	75	10–41	3.5/3	33
3D Sn/CNT-Agls	0.5 M $KHCO_3$	82.7	–	–	34
SnO_2 nanoparticles	5 M KOH	70	–	–	35
Sn nanoparticles	0.45 M $KHCO_3$ +0.5 M KCl	70	–	–	36
Sn/SnO_2 nanoparticles	0.5 M Na_2CO_3 +0.5 M Na_2SO_4	>70	26.11–68.06	–	37
SnO_2 nanoparticles	1 M $KHCO_3$	64	–	–	38
sulfur-doped	0.5 M $KHCO_3$	93	–	1	39

indium					
Bi/Bi_2O_3 nanosheets	0.5 M $KHCO_3$	~90	>50	31	40
surface-lithium-doped tin	1 M KOH	92	–	150	41
Sn/Cu HFGDE	0.5 M $KHCO_3$	78	–	4	42
Bi@Sn core-shell nanoparticles	2 M $KHCO_3$	92	56	20	43
Cu-SPy	5 M KOH	81	31	6	44
Cu_2SnS_3 nanosheets	0.5 M $KHCO_3$	83.4	–	–	45
Bi-SnO_x nanoshells	0.5 M $KHCO_3$	95.8	–	50	46
sulfur-modified Cu_2O(S3-Cu_2O-70)	0.1 M $KHCO_3$	~90	–	83	47
SiC-Nafion^{TM}/SnBi/PTFE	MEA	>90	–	125	50
Sn(S)-H	0.5 M K_2SO_4 +H_2SO_4 (pH = 3)	92.1	–	14	51
Bi nanosheets	3 M KCl +0.05 M H_2SO_4	92.2	–	8	52

Supplementary Table 3. The relative peak areas of *OCHO intermediates for Cu_6Sn_5, Cu, and Sn catalysts at the same potential range of −0.54 to −0.94 V_{RHE}. The calibrated *OCHO ratio on Cu_6Sn_5 and Sn (R1/R2) is 1.37±0.5.

Potential (V_{RHE})	*OCHO peak area on Cu_6Sn_5 calibrated using the peak areas of potassium ferricyanide as an internal standard	*OCHO peak area on Sn calibrated using the peak areas of potassium ferricyanide as an internal standard	*OCHO ratio on Cu_6Sn_5 and Sn with internal standard (R1)	*OCHO peak area on Cu_6Sn_5 without using internal standard	*OCHO peak area on Sn without using internal standard	*OCHO ratio on Cu_6Sn_5 and Sn without internal standard (R2)	R1/R2
−0.54	84.6	9.4	9.00	0.4692	0.0667	7.03	1.28
−0.64	89.3	16.5	5.41	0.5429	0.1477	3.68	1.47
−0.74	87.7	28.3	3.10	0.7188	0.3225	2.23	1.39
−0.84	94.8	40.7	2.33	1.2799	0.7348	1.74	1.34
−0.94	86.8	56.1	1.55	2.7497	2.6063	1.06	1.47

Supplementary Table 4. The proportion of Cu/Sn corresponds to Supplementary Fig. 41.

SEM-EDX	a	b	c	d	e	f	g	h
Cu: Sn	100:0	92:8	86:14	72:28	56:44	30:70	12:88	0:100

Supplementary References

1. Kresse, G. & Hafner, J. Ab initio molecular dynamics for liquid metals. *Phys. Rev. B: Condens. Matter* **47**, 558–561 (1993).

2. Hammer, B. et al. Improved adsorption energetics within density-functional theory using revised Perdew-Burke-Ernzerhof functionals. *Phys. Rev. B: Conden. Matter Mater. Phys.* **59**, 7413–7421 (1999).

3. Gajdoš, M. & Hafner, J. CO adsorption on Cu (111) and Cu (001) surfaces: Improving site preference in DFT calculations. *Surf. Sci.* **590**, 117–126 (2005).

4. Windl, W. & Chien, S.-C. Free-energy parameterization and thermodynamics in Si-Ge-Sn alloys. *Phys. Status Solidi B* **259**, 2100590 (2022).

5. Swart, JCW. et al. Surface energy estimation of catalytically relevant fcc transition metals using DFT calculations on nanorods. *JPC C.* **111**, 4998–5005 (2007).

6. Kraft, T. et al. Elastic constants of Cu and the instability of its bcc structure. *Phys. Rev. B.* **48**, 5886 (1993).

7. Zhang, D. et al. First-principles study of the structural stability of cubic, tetragonal and hexagonal phases in Mn_3Z (Z= Ga, Sn and Ge) Heusler compounds. *J. Condens. Matter Phys.* **25**, 206006 (2013).

8. Swart, JC. et al. Surface energy estimation of catalytically relevant fcc transition metals using DFT calculations on nanorods. *JPC C.* **111**, 4998–5005 (2007).

9. Foiles, SM. et al. Embedded-atom-method functions for the fcc metals Cu, Ag, Au, Ni, Pd, Pt, and their alloys. *Phys. Rev. B* **33**, 7983 (1986).

10. Xiao, H. et al. Cu metal embedded in oxidized matrix catalyst to promote CO_2 activation and CO dimerization for electrochemical reduction of CO_2. *PNAS.* **114**, 6685–6688 (2017).

11. Wang, H. et al. Oxygen reduction reaction on Pt(1 1 1), Pt(2 2 1), and Ni/Au_1Pt_3(2 2 1) surfaces: Probing scaling relationships of reaction energetics and interfacial composition. *Chem. Eng. Sci*. **184**, 239–250 (2018).

12. Malcolm, W. & Chase, Jr. NIST-JANAF thermochemical tables. Fourth edition. Washington, DC: American Chemical Society; New York: American Institute of Physics for the National Institute of Standards and Technology (1998).Chase MW.

NIST-JANAF thermochemical tables for oxygen fluorides. *JPCRD* **25**, 551–603 (1996).

13. Zhu, Y-A. et al. DFT studies of dry reforming of methane on Ni catalyst. *Catal. Today* **148**, 260–267 (2009).

14. Widom, B. Statistical mechanics: a concise introduction for chemists. Cambridge University Press (2002).

15. Nørskov, JK. et al. Origin of the overpotential for oxygen reduction at a fuel-cell cathode. *J. Phys. Chem. B* **108**, 17886–17892 (2004).

16. Tursun, M. & Wu, C. NO Electroreduction by transition metal dichalcogenides with chalcogen vacancies. *ChemElectroChem* **8**, 3113–3122 (2021).

17. Yang, F. et al. Solid-state synthesis of Cu nanoparticles embedded in carbon substrate for efficient electrochemical reduction of carbon dioxide to formic acid. *Chem. Eng. J.* **400**, 125879 (2020).

18. Mathew, K. et al. Implicit self-consistent electrolyte model in plane-wave density-functional theory. *J. Chem. Phys.* **151**, 234101 (2019).

19. Petrosyan, SA. et al. Joint density-functional theory: Ab initio study of Cr_2O_3 surface chemistry in solution. *J. Phys. Chem. B* **109**, 15436–15444 (2005).

20. Mathew, K. et al. Hennig RG. Implicit solvation model for density-functional study of nanocrystal surfaces and reaction pathways. *J. Chem. Phys.* **140**, (2014).

21. Goodpaster, JD. et al. Identification of possible pathways for C–C bond formation during electrochemical reduction of CO_2: new theoretical insights from an improved electrochemical model. *J. Phys. Chem. Lett.* **7**, 1471–1477 (2016).

22. Hormann, NG. et al. Grand canonical simulations of electrochemical interfaces in implicit solvation models. *J. Chem. Phys.* **150**, 041730 (2019).

23. Steinmann, SN. & Sautet P. Assessing a first-principles model of an electrochemical interface by comparison with experiment. *JPC C.* **120**, 5619–5623 (2016).

24. Jinnouchi, R. & Anderson AB. Aqueous and surface redox potentials from self-consistently determined Gibbs energies. *JPC C.* **112**, 8747–8750 (2008).

25. Jinnouchi, R. & Anderson, AB. Electronic structure calculations of liquid-solid interfaces: Combination of density functional theory and modified Poisson-Boltzmann

theory. *Phys. Rev. B* **77**, 245417 (2008).

26. Fan, L. et al. Electrochemical CO_2 reduction to high-concentration pure formic acid solutions in an all-solid-state reactor. *Nat. Commun.* **11**, 3633 (2020).

27. Xia, C. et al. Continuous production of pure liquid fuel solutions via electrocatalytic CO_2 reduction using solid-electrolyte devices. *Nat. Energy* **4**, 776–785 (2019).

28. Grigioni, I. et al. CO_2 Electroreduction to formate at a partial current density of 930 mA cm^{-2} with InP colloidal quantum dot derived catalysts. *ACS Energy Lett.* **6**, 79–84 (2021).

29. Zheng, T. et al. Copper-catalysed exclusive CO_2 to pure formic acid conversion via single-atom alloying. *Nat. Nanotechnol.* **16**, 1386–1394 (2021).

30. Lei, F. et al. Metallic tin quantum sheets confined in graphene toward high-efficiency carbon dioxide electroreduction. *Nat. Commun.* **7**, 12697 (2016).

31. García De Arquer, F. P. et al. 2D metal oxyhalide-derived catalysts for efficient CO_2 electroreduction. *Adv. Mater.* **30**, 1802858 (2018).

32. Chen, Y. et al. A robust, scalable platform for the electrochemical conversion of CO_2 to formate: identifying pathways to higher energy efficiencies. *ACS Energy Lett.* **5**, 1825–1833 (2020).

33. Kopljar, D. et al. Transferring electrochemical CO_2 reduction from semi-batch into continuous operation mode using gas diffusion electrodes. *Chem. Eng. Technol.* **39**, 2042–2050 (2016).

34. Chen, Z. et al. 3D hierarchical porous structured carbon nanotube aerogel-supported Sn spheroidal particles: an efficient and selective catalyst for electrochemical reduction of CO_2 to formate. *J. Mater. Chem. A* **5**, 24651–24656 (2017).

35. Löwe, A. et al. Optimizing reaction conditions and gas diffusion electrodes applied in the CO_2 reduction reaction to formate to reach current densities up to 1.8 A cm^{-2}. *ACS Sustainable Chem. Eng.* **9**, 4213–4223 (2021).

36. Del Castillo, A. et al. Sn nanoparticles on gas diffusion electrodes: Synthesis, characterization and use for continuous CO_2 electroreduction to formate. *J. CO_2 Util.* **18**, 222–228 (2017).

37. Sen, S. et al. Electroreduction of carbon dioxide to formate at high current densities

using tin and tin oxide gas diffusion electrodes. *J. Appl. Electrochem.* **49**, 917–928 (2019).

38. Liang, C. et al. High efficiency electrochemical reduction of CO_2 beyond the two-electron transfer pathway on grain boundary rich ultra-small SnO_2 nanoparticles. *J. Mater. Chem. A* **6**, 10313–10319 (2018).

39. Ma, W. et al. Promoting electrocatalytic CO_2 reduction to formate via sulfur-boosting water activation on indium surfaces. *Nat. Commun.* **10**, 892 (2019).

40. Wu, D. et al. Boosting formate production at high current density from CO_2 electroreduction on defect-rich hierarchical mesoporous Bi/Bi_2O_3 junction nanosheets. *Appl. Catal. B: Environ.* **271**, 118957 (2020).

41. Yan, S. et al. Electron localization and lattice strain induced by surface lithium doping enable ampere level electrosynthesis of formate from CO_2. *Angew. Chem. Int. Ed.* **60**, 25741–25745 (2021).

42. Rabiee, H. et al. Tuning the product selectivity of the Cu hollow fiber gas diffusion electrode for efficient CO_2 reduction to formate by controlled surface Sn electrodeposition. *ACS Appl. Mater. Interfaces* **12**, 21670–21681 (2020).

43. Xing, Y. et al. Bi@Sn core–shell structure with compressive strain boosts the electroreduction of CO_2 into formic acid. *Adv. Sci.* **7**, 1902989 (2020).

44. Creissen, C. E. et al. Molecular inhibition for selective CO_2 conversion. *Angew. Chem. Int. Ed.* **61**, e202206279 (2022).

45. Wang, W. et al. In situ phase separation into coupled interfaces for promoting CO_2 electroreduction to formate over a wide potential window. *Angew. Chem. Int. Ed.* **60**, 22940–22947 (2021).

46. Yang, Q. et al. Novel Bi-doped amorphous SnO_x nanoshells for efficient electrochemical CO_2 reduction into formate at low overpotentials. *Adv. Mater.* **32**, 2002822 (2020).

47. Ma, X. et al. Facet dopant regulation of Cu_2O boosts electrocatalytic CO_2 reduction to formate. *Adv. Funct. Mater.* **33**, 2213145 (2023).

48. Xu, Y. et al. Self-cleaning CO_2 reduction systems: Unsteady electrochemical forcing enables stability. *ACS Energy Lett.* **6**, 809–815 (2021).

49. Kim, C. et al. Impact of pulsed electrochemical reduction of CO_2 on the formation of C_{2+} products over Cu. *ACS Catal.* **10**, 12403–12413 (2020).

50. Li, L. et al. Achieving high single-pass carbon conversion efficiencies in durable CO_2 electroreduction in strong acids via electrode structure engineering. *Angew. Chem. Int. Ed.* **62**, e202300226 (2023).

51. Shen, H. et al. Acidic CO_2-to-HCOOH electrolysis with industrial-level current on phase engineered tin sulfide. *Nat. Commun.* **14**, 2843 (2023).

52. Qiao, Y. et al. Engineering the local microenvironment over Bi nanosheets for highly selective electrocatalytic conversion of CO_2 to HCOOH in strong acid. *ACS Catal.* **12**, 2357–2364 (2022).
